# Atf3 controls transitioning in female mitochondrial cardiomyopathy as identified by spatial and single-cell transcriptomics

**DOI:** 10.1126/sciadv.adq1575

**Published:** 2025-04-04

**Authors:** Tasneem Qaqorh, Yusuke Takahashi, Kohei Sameshima, Kentaro Otani, Issei Yazawa, Yuya Nishida, Kohei Tonai, Yoshitaka Fujihara, Mizuki Honda, Shinya Oki, Yasuyuki Ohkawa, David R. Thorburn, Ann E. Frazier, Atsuhito Takeda, Yoshihiko Ikeda, Heima Sakaguchi, Takuya Watanabe, Norihide Fukushima, Yasumasa Tsukamoto, Naomasa Makita, Osamu Yamaguchi, Kei Murayama, Akira Ohtake, Yasushi Okazaki, Takanari Kimura, Hisakazu Kato, Hijiri Inoue, Ken Matsuoka, Seiji Takashima, Yasunori Shintani

**Affiliations:** ^1^ Department of Molecular Pharmacology, National Cerebral and Cardiovascular Center, Suita, Osaka, Japan.; ^2^ Department of Medical Biochemistry, Osaka University Graduate School of Frontier Biosciences, Suita, Osaka, Japan.; ^3^ Department of Advanced Medical Technologies, National Cerebral and Cardiovascular Center, Suita, Osaka, Japan.; ^4^ Department of Drug Discovery Medicine, Kyoto University Graduate School of Medicine, Kyoto, Japan.; ^5^ Division of Transcriptomics, Medical Institute of Bioregulation, Kyushu University, Fukuoka, Japan.; ^6^ Murdoch Children’s Research Institute, Royal Children’s Hospital, and University of Melbourne, Department of Paediatrics, Parkville, Victoria, Australia.; ^7^ Victorian Clinical Genetics Services, Royal Children’s Hospital, Parkville, Victoria, Australia.; ^8^ Department of Pediatrics, Faculty of Medicine, Hokkaido University, Sapporo, Japan.; ^9^ Department of Pathology, National Cerebral and Cardiovascular Center, Suita, Osaka, Japan.; ^10^ Department of Pediatric Cardiology, National Cerebral and Cardiovascular Center, Suita, Osaka, Japan.; ^11^ Department of Transplant Medicine, National Cerebral and Cardiovascular Center, Suita, Osaka, Japan.; ^12^ Senri Kinran University, Suita, Osaka, Japan.; ^13^ Omics Research Center, National Cerebral and Cardiovascular Center, Suita, Osaka, Japan.; ^14^ Department of Cardiology, Sapporo Teishinkai Hospital, Sapporo, Japan.; ^15^ Department of Metabolism, Chiba Children’s Hospital, Chiba, Japan.; ^16^ Diagnostics and Therapeutic of Intractable Diseases, Intractable Disease Research Center, Graduate School of Medicine, Juntendo University, Tokyo, Japan.; ^17^ Department of Pediatrics and Clinical Genomics, Saitama Medical University, Moroyama, Saitama, Japan.

## Abstract

Oxidative phosphorylation defects result in now intractable mitochondrial diseases (MD) with cardiac involvement markedly affecting prognosis. The mechanisms underlying the transition from compensation to dysfunction in response to metabolic deficiency remain unclear. Here, we used spatially resolved transcriptomics and single-nucleus RNA sequencing (snRNA-seq) on the heart of a patient with mitochondrial cardiomyopathy (MCM), combined with an MCM mouse model with cardiac-specific Ndufs6 knockdown (FS6KD). Cardiomyocytes demonstrated the most heterogeneous expression landscape among cell types caused by metabolic perturbation, and pseudotime trajectory analysis revealed dynamic cellular states transitioning from compensation to severe compromise. This progression coincided with the transient up-regulation of a transcription factor, *ATF3*. Genetic ablation of *Atf3* in FS6KD corroborated its pivotal role, effectively delaying cardiomyopathy progression in a female-specific manner. Our findings highlight a fate-determining role of *ATF3* in female MCM progression and that the latest transcriptomic analysis will help decipher the mechanisms underlying MD progression.

## Teaser

Transcriptomics of human and mouse cardiomyopathy hearts identified a crucial factor in the disease progression.

## INTRODUCTION

Abnormalities in oxidative phosphorylation (OXPHOS) lead to metabolic insufficiencies resulting in systemic mitochondrial diseases (MDs). Congenital mitochondrial disorders can arise from mutations in mitochondrial DNA (mtDNA) or genomic DNA (*1*). As numerous gene products contribute to mitochondrial integrity and OXPHOS homeostasis, more than 400 causative genes have been identified (*2*). This genetic diversity contributes to the high prevalence and heterogeneity of MDs, affecting about 1 in 4000 individuals (*3*, *4*). Moreover, emerging evidence highlights tissue-specific (*5*) and even cell-specific (*6*) responses. It should be noted that there are compensatory mechanisms for dysfunctional or damaged mitochondria, including mtDNA repair, mitochondrial dynamics (fission and fusion), and increased mitochondrial biogenesis (*7*, *8*). However, insults exceeding cellular compensatory capacity drive dysfunction, leading to tissue failure and disease manifestation. Mechanisms underlying the transition from compensation to dysfunction in disease progression remain unknown. These complexities, coupled with an incomplete understanding of molecular mechanisms of disease progression, have hindered therapeutic development, and consequently MD remains intractable.

Previous studies have elucidated stress response pathways activated by mitochondrial dysfunction, including the mitochondrial unfolded protein response and integrated stress response (ISR^mt^) (*9*). ISR^mt^ triggers eukaryotic translation initiation factor 2 alpha phosphorylation through upstream cascades, suppressing global translation and selectively inducing downstream players, such as activating transcription factor 4 (Atf4), Atf5, DNA damage–inducible transcript 3, C/EBP homologous protein (CHOP), and fibroblast growth factor 21 (Fgf21) (*10*–*17*). Although ISR^mt^ is acknowledged as a critical modulator of MD progression, its role is context or model dependent (*16*–*18*). ISR^mt^ may primarily serve as a protective response for cellular recovery, yet prolonged ISR^mt^ could trigger detrimental effects. Even when ISR^mt^ plays a protective role, accelerated tissue dysfunction persists despite the inhibition of ISR^mt^-associated mediators and downstream players, suggesting that the transition from compensation to dysfunction is decided before ISR^mt^ activation (*14*, *18*). However, what triggers the disease transition in MD is unknown.

MD is a multiorgan disorder, and cardiac involvement is particularly a risk factor for poor prognosis (*19*–*21*). Mitochondrial cardiomyopathy (MCM) is now garnering research attention (*22*), and mitochondrial deficiency can manifest independently in the heart. In either case, multiorgan or just cardiac, no specific treatment for MCM is available; options are limited to general heart failure therapy. Diagnosis of MCM is challenging, especially for nonsystemic MCM cases. Light microscopy often shows vacuolar change or increased mitochondrial aggregates, but these signatures can be seen in other cardiomyopathies. Enzyme activity measurement, quantitative histopathological assessment of respiratory chain (RC) complex, and genetic screening are usually required, but the diagnosis rate is limited (*23*). Histological assessment of MCM tissues often shows heterogeneity in the myocardium, which contains cardiomyocytes with less vacuolar change and ones with severe vacuolar change, in addition to fibrosis, probably suggesting a mixture of disease states; however, it remains elusive.

Recently, spatially resolved transcriptomic technology has emerged as a useful research tool that can be applied to clinical specimens, maintaining topological information (*24*). We used spatial transcriptomics and single-nucleus RNA sequencing (snRNA-seq) on the heart of a patient with MCM collected during ventricular assist device implantation to gain insights into the molecular mechanisms underlying MD progression. *ATF3* was distinctively expressed in less-damaged cardiomyocytes before transitioning to failing cardiomyocytes. We verified our findings by snRNA-seq analysis of heart samples collected at different time points from an MCM mouse model with cardiac-specific *Ndufs6* knockdown (FS6KD) (*25*). The genetic ablation study further corroborated the pivotal role of *Atf3* in female MCM progression.

## RESULTS

### Cardiomyocytes of a human patient with complex IV deficiency display transitioning to diseased state

**Fig. 1.**
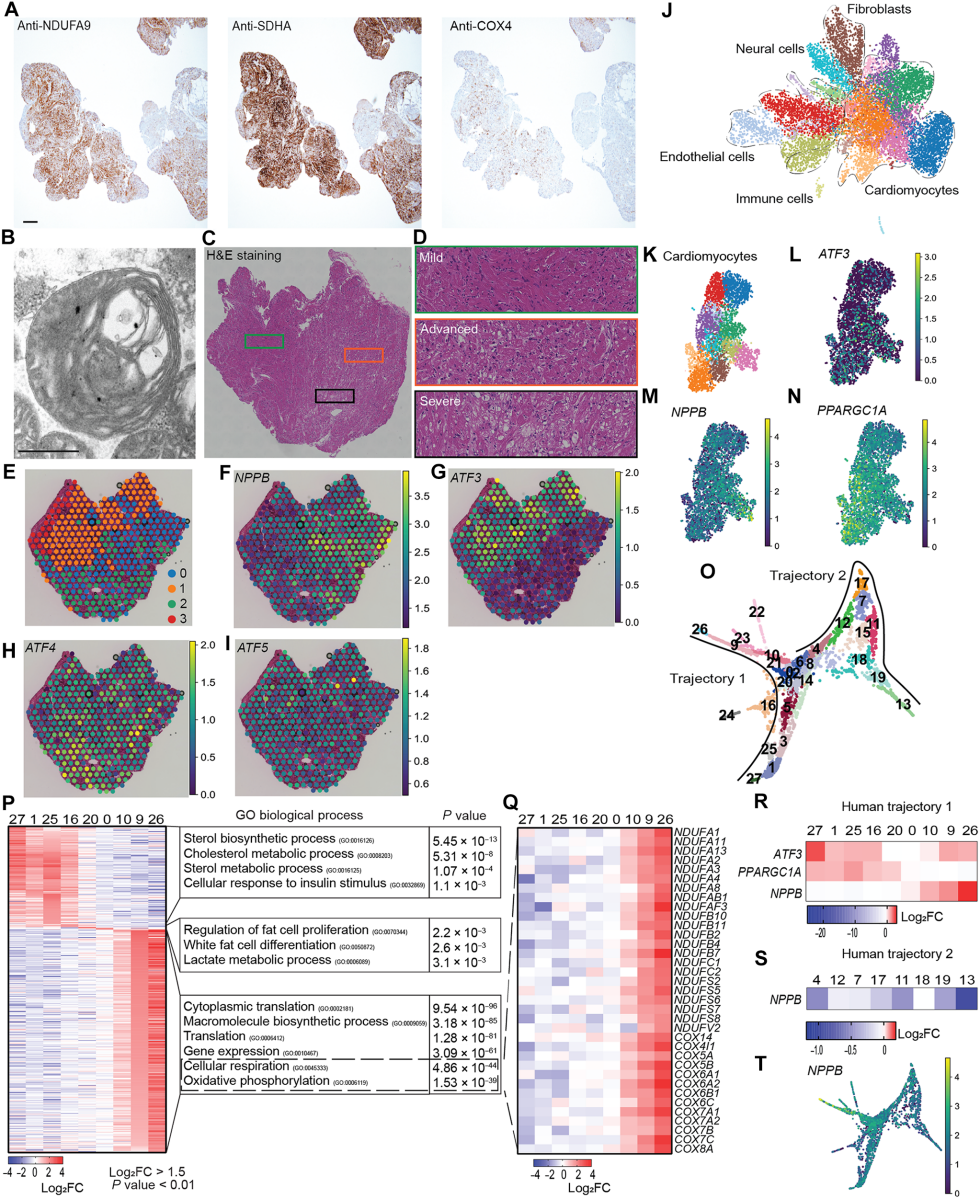
Cardiomyocytes of a patient with MCM demonstrated transitioning. (**A**) Immunohistochemical assessment of RC complexes abundance on myocardium from the female 9-month MCM patient. Immunostaining with NDUFA9 antibody (complex I, top), SDHA antibody (complex II, middle), and COX4 antibody (complex IV, bottom. Scale bar, 200 μm. (**B**) Electron microscopy ultrastructure of abnormal cardiac mitochondria showing swelling and abnormal onion-like concentric membranes. Scale bar, 500 nm. (**C** and **D**) H&E staining of left ventricle showing three phenotypically heterogeneous regions of (D) mild (top; green box), advanced (middle; orange box), and severe (bottom; black box) tissue dysfunction areas. (**E**) Unsupervised clustering of expression profiles for spots (*n* = 488) onto the tissue section. (**F** to **I**) Expression features of (F) *NPPB*, (G) *ATF3*, (H) *ATF4*, and (I) *ATF5* shown as spots. (**J**) UMAP plot of snRNA-seq data of cardiac cellular populations (*n* = 10,868) from the patient with MD colored by Leiden clusters and annotated. (**K** to **N**) UMAP plot of isolated cardiomyocytes (*n* = 3511) (K) colored by Leiden clusters. Expressions of (L) *ATF3*, (M) *PPARGC1A*, and (N) *NPPB* are shown on the UMAP. (**O**) ForceAtlas2 (FA2) scatterplot of PAGA trajectory from cardiomyocytes numbered by Leiden cellular states, showing two trajectories. (**P** to **S**) Heatmaps displaying (P) top up-regulated genes in the cellular states of trajectory 1 ranked by log_2_FC and associated top GO terms and (Q) expression levels of complex I and complex IV subunits and assembly factors extracted from trajectory 1, (R) *ATF3* expression level changes in comparison to *PPARGC1A* and *NPPB*, and (S) *NPPB* along the trajectory 2 states. (**T**) *NPPB* expression shown as FA2 scatterplot.

To comprehend disease development mechanisms, we conducted spatially resolved transcriptomics and snRNA-seq analysis on a small ventricular tissue sample from a 9-month-old female patient with MCM who exhibited severe complex IV reduction indicated by cytochrome c oxidase subunit 4 (COX4) staining and characteristic abnormal cristae observed via electron microscopy (Fig. 1, A and B). Cardiac tissues were collected during ventricular assist device implantation as the patient suffered severe cardiac failure. We assessed the tissue heterogeneity before sequencing with hematoxylin and eosin (H&E) staining. Albeit the severe heart failure manifested at the time of tissue collection, the heart tissue showed wide heterogeneity: the areas with relatively healthy cardiomyocytes and mild fibrotic injury (Fig. 1, C and D; referred to as mild), areas with more damaged cardiomyocytes containing vacuoles (referred to as advanced), and areas with severely damaged cardiomyocytes with large vacuoles and the highest degree of fibrosis (referred to as severe). With this pathological assessment, it is conceivable that the myocardium progresses in this order along the disease course; however, nonsynchronous progression in each part resulted in the tissue heterogeneity observed in the section. Subsequently, we applied spatially resolved transcriptomics using the same heart section. Unsupervised clustering revealed different clusters corresponding to each area in the H&E staining (Fig. 1E), corroborating the tissue heterogeneity. Next, we assessed the expression of natriuretic peptide B (*NPPB*) (*26*), a well-established biomarker of heart failure (Fig. 1F). Unexpected to our assumption that the highest *NPPB* expression would be found in the severe area, *NPPB* expression was the highest in the advanced area. It might indicate a reduced number of cardiomyocytes. We performed a deconvolution analysis that decomposed estimated gene count matrices for each cell type per spot using the Cell2location (*27*) deconvolution model. However, cardiomyocytes’ spatial distribution was not lower in the severe region compared to the advanced (fig. S1A). Therefore, lower *NPPB* expression levels in the severe area could be due to negative feedback or exhaustion. Alternatively, intercellular communication might have affected *NPPB* expression in cardiomyocytes, considering that fibroblasts, myeloid, lymphoid, and adipocytes also have greater abundance in the severe area (fig. S1A).

Notably, we identified one transcription factor (TF), activating transcription factor 3 (*ATF3*), from the differentially expressed genes (DEGs) analysis up-regulated in the mild areas (cluster 1) of the section (Fig. 1, E and G). *ATF3* expression was strong in spots in the mild areas and weak or faded as the tissue transitioned to damage, contrasting to *NPPB* expressing areas. *ATF3* was a weak but negatively correlated gene with *NPPB* and one of the top five negatively correlated genes with *NPPA*, another heart failure marker, and a neighbor gene of *NPPB* (fig. S1A). *ATF3* is a member of the ATF/cAMP response element–binding protein (CREB) TF family that has been extensively studied for its roles in ISR^mt^ upon mitochondrial dysfunction, primarily *ATF4* and *ATF5* (*10*, *17*, *18*). In contrast, *ATF3* has received less attention and is generally considered a minor downstream target of *ATF4* or *ATF5* (*16*). However, our spatial transcriptomics revealed that the expression of *ATF4* and *ATF5* did not contrast to *ATF3* (mild area), relate with *NPPB* (advanced) (Fig. 1, H and I), or did not show a clear correlation in the Spearman test (fig. S1, A and B); rather, their expression corresponded to regions already showing more fibrosis and damage, the severe area. The data suggest that *ATF3* up-regulation might have a distinct role other than *ATF4* and *ATF5*, possibly in the disease transition in the patient with MCM.

Spots in the gene expression slide are 55 μm in diameter, thus possibly representing expression from multiple cells with different cell types under each spot. To identify which cell types express *ATF3*, we performed a snRNA-seq analysis on the same tissue collected during ventricular assist device implantation. Unsupervised clustering revealed multiple cellular populations (Fig. 1J). *ATF3* was expressed dominantly in cardiomyocytes, while *ATF4* and *ATF5* are expressed in other cell types, including immune cells and fibroblasts (fig. S1, C to E). The expression of *ATF3* and *NPPB* was heterogeneous in cardiomyocytes (Fig. 1K). Cardiomyocytes with higher *NPPB* expression showed relatively weak *ATF3* expression (right bottom in Fig. 1, L and M) and vice versa (left bottom in Fig. 1, L and M), confirming our findings in the spatial analysis albeit less impressive. Notably, peroxisome proliferator–activated receptor gamma coactivator 1-alpha (*PPARGC1A*), which was one of the top genes correlated with *ATF3* in the spatial analysis (Fig. 1N and fig. S1A), demonstrated a clear contrasting expression pattern to *NPPB. PPARGC1A* is a transcription coactivator and mitochondrial biogenesis master regulator (*28*) that is up-regulated in cells as a compensatory response to increased energy needs or mitochondrial dysfunction.

The advantage of snRNA-seq, which contains a greater number of cells with higher heterogeneity than the spatial analysis, along with the presence of *NPPB* as a marker for heart failure, led us to use pseudotime trajectory inference (*29*) analysis to investigate ongoing dynamic processes within the tissue. We identified a trajectory composed of two branches with two terminals (Fig. 1O); we called trajectory 1 and trajectory 2. We denoted state 27 as the root state based on high *ATF3* and *PPARGC1A* expressions with low *NPPB* expression. Gene ontology (GO) enrichment analysis of early human states revealed metabolic regulation processes (Fig. 1P), whereas late trajectory 1 states featured enrichment of aerobic respiration pathways and energy production, particularly in states 9 and 26, with complex I and IV subunits and assembly factors highly expressed (Fig. 1Q). In contrast, the DEGs in trajectory 2 did not highlight mitochondria-related genes (fig. S1F). One notable GO is DNA damage response, which was up-regulated in state 11 in trajectory 2. DNA damage plays a critical role in the development of human and murine model of heart failure (*26*, *30*–*34*). DNA damage signatures are reported as an indicator of cardiomyocytes that are irreversibly damaged. Considering that cardiomyocytes in trajectory 2 showed decreased *NPPB* expression (Fig. 1, R to T), it is likely that these cardiomyocytes had already passed the point of no return, leaving no room for therapeutic intervention. Thus, we wanted to focus on the *ATF3*-containing trajectory 1 hereafter.

High-energy demanding tissues show enrichment of *PPARGC1A* under normal physiological conditions (*35*). To ascertain that its expression in mild states is specific to the context of the disease, we integrated the MCM dataset with snRNA-seq data obtained from healthy cardiomyocytes sourced from the heart of an 11-year-old female donor (*36*). The cardiomyocytes from the healthy donor manifested distinct clustering patterns and did not demonstrate heightened expression of *PPARGC1A*, *ATF3*, or *NPPB* (fig. S1, G to J). These data validate that the observed gene up-regulation is not an inherent phenotype in human heart tissue. Overall, our MCM transcriptome analysis revealed a higher level of heterogeneity within MCM tissue that may provide insights into the disease progression mechanism. *ATF3*-positive cardiomyocytes in trajectory 1 in snRNA-seq and in mild area in the spatial analysis represent less damaged cardiomyocytes that may still hold room for therapeutic intervention, and the TF *ATF3* might play a role in disease transition.

### MCM mouse model shows up-regulation of *Ppargc1a* preceding cardiomyocyte dysfunction

Transcriptome analysis on the heart from the patient with MCM revealed a high level of heterogeneity and a possible important role of *ATF3* in disease progression. However, we only analyzed one patient at a single time point, which casts doubt on the extrapolation of our findings considering that MDs have more than 400 causative genes, likely having vast diversity in phenotypes and disease progression mechanisms. Moreover, one of the hurdles in studying early factors in MCM progression is the uncontrolled experimental settings, such as the availability of specimens before the phenotypic manifestation of the disease. Therefore, we used the murine MCM model to validate our findings.

Among several murine MCM models previously reported, it was ideal to assess a slow progressing one to dissect disease transitioning. We used an MCM model with heart-dominant FS6KD. Ndufs6 is a subunit of complex I in the mitochondrial RC. Although the diseased RC complex is different from the patient with MCM we analyzed, decreased complex I activity is the predominant defect among patients with MD (*37*–*40*), and this model allows for noninvasive cardiac function measurement as the mice can survive longer than other models. FS6KD mice showed good general health status up until 4 months old for males and 8 months old for females. They start showing sudden death due to heart failure and do not often show clear manifestation of symptoms [e.g., body weight (BW) reduction]. We found that the female FS6KD mice exhibited slow progression, evident by a gradual decline in left ventricular ejection fraction (EF) beginning at 6 to 8 weeks (fig. S2, A and B), while male mice exhibited a lower left ventricular EF% beginning at an early age and rapidly develop cardiac failure (*25*). Left ventricular dilatation, measured as left ventricular internal dimension in diastole (LVDd), is also a fate-determining factor. Female FS6KD mice showed progressive LV dilatation from 8 to 17 weeks old (fig. S2A). Enzymology analysis showed no difference in the extent of decreased complex I activity between males and females (fig. S2C). Yet, female FS6KD mice showed milder phenotype (disease progression, the speed of decline in cardiac function, or survival), suggesting that it rather depends on the downstream response to complex I deficiency. We reasoned that female mice would be suitable for dissecting MCM transitioning; thus, we collected female heart tissues of FS6WT and FS6KD mice at different disease stages extracted from 8-week (early-stage) FS6KD and 17-week (late-stage) FS6KD for snRNA-seq analysis.

**Fig. 2.**
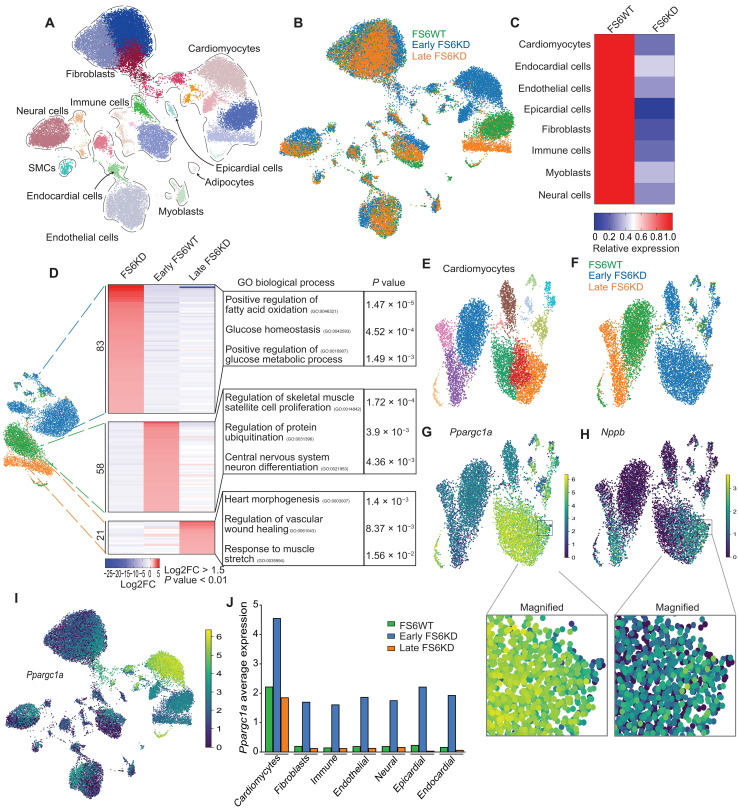
Mouse MCM model shows earlier cardiomyocyte metabolic compensation. (**A** and **B**) UMAP plot of the cardiac cellular populations in the integrated dataset. (A) Colored by Leiden clusters and annotated. (B) Colored by sample identity, FS6WT (green, *n* = 12,020), early FS6KD (blue, *n* = 12,516), and late FS6KD (orange, *n* = 7280). (**C**) Heatmap displaying knockdown efficiency per cell type. Relative average expression levels of *Ndufs6* in cell types of early FS6KD against FS6WT. (**D**) UMAP plot of isolated cardiomyocytes (FS6WT: *n* = 2856, early FS6KD: *n* = 4273, late FS6KD: *n* = 1201) colored by sample identity with respective heatmaps displaying top up-regulated genes ranked by log_2_FC and associated enriched top GO terms. Fisher exact test was used to calculate *P* values for GO. (**E** and **F**) UMAP plot of isolated and subclustered cardiomyocytes. (E) Colored by Leiden clusters. (F) Colored by sample identity. (**G**) *Ppargc1a* expression shown as isolated cardiomyocyte UMAP feature. Small subcluster from early FS6KD was magnified and shown in the bottom highlighting decreased expression. (**H**) *Nppb* expression shown as isolated cardiomyocytes UMAP feature. Small subcluster from early FS6KD was magnified and shown in the bottom highlighting increased expression. (**I**) *Ppargc1a* expression shown as integrated dataset UMAP feature. Color intensity is increased in cellular populations corresponding to early FS6KD in all cell types. (**J**) *Ppargc1a* average expression per cell type per dataset. SMCs, smooth muscle cell.

Unsupervised clustering of integrated datasets revealed distinct cardiac cell populations, including cardiomyocytes, fibroblasts, immune cells, and minor populations, such as myoblasts (Fig. 2A and fig. S2D). Cellular composition variations were evident, with cardiomyocytes increasing and fibroblasts decreasing notably in early-stage FS6KD hearts, and this change was inverted in late-stage FS6KD hearts (fig. S2E). Notably, both epicardial and endocardial populations decreased in FS6KD, whereas adipocytes were exclusive to the wild type (WT; fig. S2F). Metabolic dysfunction prompted cellular state shifts, most prominently in cardiomyocytes, as observed in disease stage-specific clusters (Fig. 2B). FS6KD levels were similar across cell types (Fig. 2C), indicating inherent cell-specific responses driving the observed shifts. Cardiomyocyte transcriptome assessed by DEG analysis revealed dynamic changes in metabolic regulation at early stage, including increased fatty acid oxidation (FAO) and glycolytic shift, as reflected in significant GOs (Fig. 2D). The genes marked at early-stage were down-regulated at late-stage, whereas genes often associated with heart failure were up-regulated at late stage, with up-regulation in genes enriched in response to muscle stretch GO terms (Fig. 2D). These suggest that up-regulation of processes in response to metabolic deficiency in early-stage cardiomyocytes failed to halt disease progression. Analysis of isolated and subclustered cardiomyocytes (Fig. 2, E and F) showed that the most significant DEG in the early stage was *Ppargc1a* (Fig. 2G). Conversely, *Nppb* was up-regulated in late-stage cardiomyocytes (Fig. 2H). We compared and integrated two snRNA-seq datasets from 8-week and 17-week FS6WT to confirm that these differences were not driven by aging. The clustering revealed no shifting of any cell types, particularly cardiomyocytes (fig. S2G). The existence of epicardial cells, endocardial cells, and adipocytes was unaffected in 17-week FS6WT mice (fig. S2H; in orange). *Ppargc1a*, *Atf3*, *Nppb*, or *Ankrd1* expression did not change significantly between 8-week and 17-week FS6WT cardiomyocytes, nor did any of the other genes we observed differentially expressed in FS6KD cardiomyocytes (fig. S2G). In addition, we have compared the expression of the genes listed as hallmarks of aging (CellAge database; https://genomics.senescence.info/cells/index.html), including the genes involved in cellular senescence, mitochondrial function, telomere maintenance, etc. No change was observed in these genes’ expression between 8- and 17-week WT (fig. S2H). These data suggest that aging has not played a substantial role during the period we analyzed in these female mice, and the disease transition observed in trajectory analysis in female FS6KD mice was not primarily affected by aging. The snRNA-seq of a 10-week male FS6WT heart likewise integrated with the 8-week FS6WT without shifting (fig. S2I), suggesting that the results we found in the MCM mice are solely due to the knockdown of *Ndufs6*.

Similar to snRNA-seq from the MCM patient’s heart, *Ppargc1a* was markedly attenuated in the late-stage cardiomyocyte cluster (Fig. 2, G and I). We also visualized a small subcluster of the early-stage cardiomyocytes with down-regulated levels of *Ppargc1a* and up-regulation of *Nppb*, resembling late-stage cardiomyocytes (Fig. 2, G and H; magnified bottom). These findings suggest that transitioning from 8-week cardiomyocytes toward a maladaptive state is also conserved in mice. Other cardiac cell responses to metabolic deficiency showed consistent *Ppargc1a* up-regulation at early stage across all cell types, with pronounced elevation in cardiomyocytes (Fig. 2J). Moreover, expression levels were restored to baseline in all cell types when progressed to late stage. Under normal conditions, cardiomyocytes exhibited a higher baseline of *Ppargc1a* expression, indicating their heightened metabolic demands and the probable susceptibility to metabolic perturbations.

### Mouse pseudotime trajectory analysis reveals *Atf3* induction in MCM transition

**Fig. 3.**
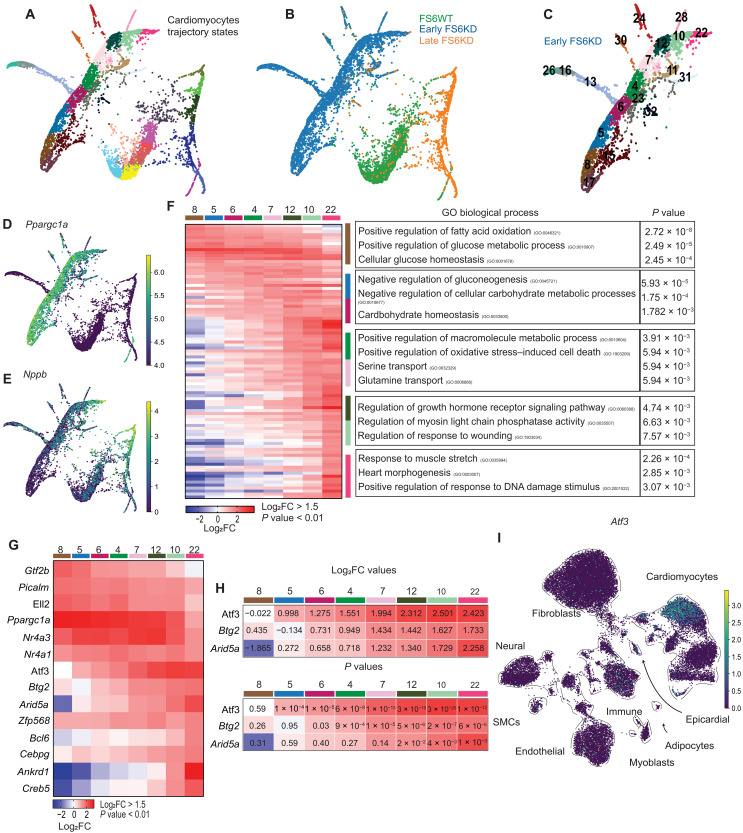
Pseudotime trajectory analysis revealed *Atf3* induction during transitioning. (**A** and **B**) FA2 scatterplot of abstracted partition-based graph abstraction (PAGA) trajectory from isolated cardiomyocytes. (A) Colored by Leiden cellular states. (B) Colored by sample identity. (**C**) FA2 scatterplot of isolated PAGA trajectory from early FS6KD cardiomyocytes numbered by Leiden cellular states. (**D** and **E**) *Ppargc1a* (D) and *Nppb* (E) expression shown as FA2 scatterplot. (**F**) Heatmap displaying top up-regulated genes in the cellular states of early FS6KD cardiomyocyte trajectory ranked by log_2_FC and associated top GO terms. Fisher exact test was used to calculate *P* values for GO. (**G**) Heatmap displaying expression levels of TFs extracted from (F) ranked by log_2_FC. (**H**) Enlarged heatmap from (G) with log_2_FC values (top) and *P* values (bottom). Wilcoxon rank-sum test was used to calculate *P* values. (**I**) *Atf3* expression shown as integrated dataset UMAP feature. Color intensity is increased in early FS6KD cardiomyocytes.

We identified a trajectory composed of eight consecutive cellular states (Fig. 3, A to C) in early-stage FS6KD cardiomyocytes dynamically transitioning from compensatory to maladaptive states. *Ppargc1a* expression consistently decreased through the trajectory to state 22 (Fig. 3D), while *Nppb* expression increased in state 22 and late-stage FS6KD (Fig. 3E). States 8 through 6 exhibited enrichment of metabolic regulatory processes switching to glycolytic metabolism (Fig. 3F), akin to GOs found in early-stage FS6KD cardiomyocytes (Fig. 2D). GO terms associated with reduced heart function were observed in states 10 and 22, corresponding to late-stage FS6KD cardiomyocytes ontology. Thus, we labeled states 8 and 22 as early and late states, paralleling early and late stages, respectively. Early-state cardiomyocytes (states 8 and 5) did not show up-regulation of *Nppb* or other heart failure–related genes but exhibited high *Ppargc1a* expression levels, suggesting compensation for mitochondrial dysfunction (Fig. 3, C to E). Considering the similarity between the late states in early-stage trajectory and late-stage cardiomyocytes, we hypothesized that the cellular transition from compensation to dysfunction occurs in early-stage cardiomyocytes, with genes expressed during mid-trajectory states (states 4 through 12) influencing cellular fate transition.

In the MCM patient’s heart, we identified *ATF3* as a possible transitioning factor. *Atf3* was among the up-regulated DEGs extracted from cardiomyocytes, specifically in 8 weeks. *Atf3* was up-regulated in mid-trajectory states 4 and 7 of early-stage FS6KD mouse (Fig. 3G) confirming our hypothesis, showing a higher fold change expression value and a lower *P* value (Fig. 3H) compared to *Btg2* that was the only other gene expressed in the same states. In addition, *Atf3* is a member of the ATF family that functions as a stress response factor. In contrast, *Btg2* is an immediate early gene induced by growth factors, and its function has been linked to cell cycle regulation or cardiac hypertrophy (*41*, *42*), a less likely candidate for transition factor in MCM. Moreover, *Atf3* was up-regulated in early-stage cardiomyocytes, down-regulated in late-stage cardiomyocytes, and not expressed in other cell types or WT cardiomyocytes (Fig. 3I). Earliest state in mouse trajectory showed that *Ppargc1a* high levels did not express *Atf3* (Fig. 3G, state 8), different from the human trajectory states showing *Atf3* expressed starting the earliest state (Fig. 1R, state 27). However, given that mouse mid to late states show coexpression of them, we presumed that the human state 27 in Fig. 1R corresponds to the mid to late states in the early-stage cardiomyocyte trajectory, having already passed the early state. These findings present strong evidence that *Atf3* acts as a transcriptional regulator of cardiomyocytes, influencing their fate toward dysfunction.

### Cluster of Atf3-expressing cardiomyocytes with repressed OXPHOS genes in the FS6KD heart

**Fig. 4.**
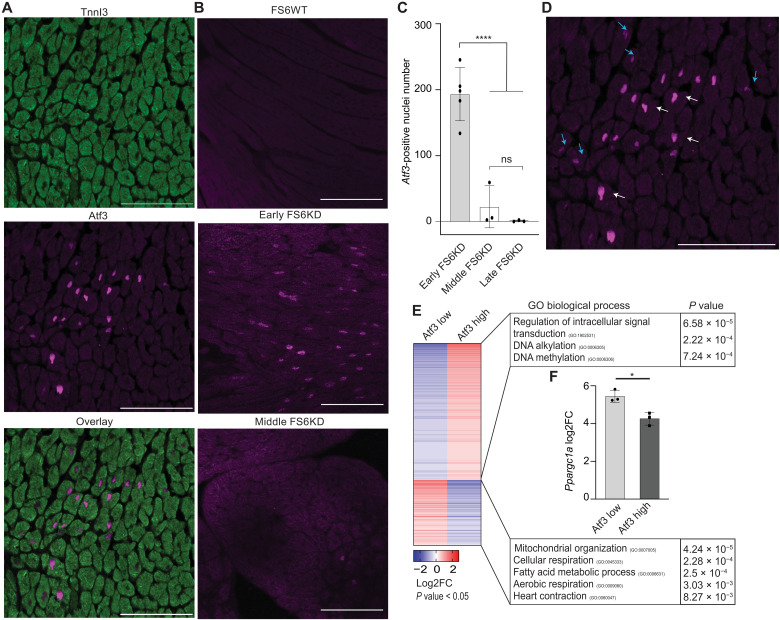
Atf3-expressing cardiomyocytes with repressed OXPHOS genes form cluster in the FS6KD heart. (**A**) Cardiomyocytes nuclear localization of Atf3 in early FS6KD. Immunostaining with TnnI3 antibody (top), Atf3 antibody (middle), and an overlay image (bottom). Scale bars, 100 μm. A representative image was shown (*n* = 11 biological replicates and *n* > 3 technical replicates). (**B**) Expression and nuclear localization of Atf3 in FS6WT versus FS6KD of different ages. Immunostaining with Atf3 antibody in FS6WT (top), early FS6KD (middle), and middle FS6KD (bottom). Scale bars, 100 μm. (**C**) Quantitative analysis of Atf3-positive nuclei by immunostaining in FS6KD of different stages (early, middle, and late). Bars: Means ± SD. Dots: Each dot represents one mouse and average count (early *n* = 5, middle *n* = 3, and late *n* = 3 biological replicates and *n* > 3 technical replicates per group). Ordinary one-way analysis of variance (ANOVA) and Tukey’s test for multiple comparisons, statistical significance: ****P ≤ 0.0001; ns, not significant. (**D**) Atf3 expression cluster from [(A), middle]. Blue arrows point to nuclei with low Atf3 expression, and white arrows point to nuclei with high Atf3 expression. Scale bar, 100 μm. (**E**) Heatmap displaying DEGs extracted from photo-isolation chemistry ranked by log_2_FC and associated top GO terms, *n* = 3 biological replicates per group. Pairwise two-tailed *t* test was used to calculate *P* values for DEG selection, and Fisher exact test was used to calculate *P* values for GO. (**F**) *Ppargc1a* expression levels. Bars: Means ± SD. Dots: Individual subjects (*n* = 3 biological replicates per group). Pairwise two-tailed *t* test, statistical significance: *P ≤ 0.05.

To validate our findings obtained from the trajectory analysis of snRNA-seq, we conducted a protein-based and spatiotemporal assessment of the FS6KD heart. Immunostaining analysis of Atf3 in FS6KD cardiac tissues showed that most of the Atf3-expressing cells were cardiomyocytes, positive for the cardiomyocyte-specific marker TnnI3 (Fig. 4A), consistent with the snRNA-seq results. Quantitative assessment confirmed that Atf3 expression was generally high in young FS6KD mice (Fig. 4, B and C), not observed in WT or older FS6KD mice. Atf3-positive signals were observed in clusters across cardiac sections, varying in intensity. Central nuclei exhibited higher signal intensity of the surroundings, indicating an expression gradient (Fig. 4D). We hypothesized that *Ppargc1a* expression would be lower in nuclei with higher Atf3 levels and vice versa. Using photo-isolation chemistry (*43*, *44*), an immunostaining-based method capable of extracting transcriptome information from locally defined areas by photo-irradiation, we separated the transcriptomes of cardiomyocytes with high or low Atf3 expression levels within Atf3-positive clusters (fig. S3A). GO analysis of gene expression profiles in cells with high Atf3 expression levels revealed increased metabolic dysfunction, impaired respiration, and reduced cardiac contractility (Fig. 4E). Cells with high Atf3 expression displayed reduced *Ppargc1a* expression compared to cells with low Atf3 expression (Fig. 4F).

Our observation in the FS6KD mice was that the compensatory mechanism is active in the early stage; however, it turned off in the late stage (Fig. 2, D and G). Atf3 can repress or activate target genes by binding to a consensus DNA sequence (5′-TGACGTCA-3′) via its basic region-leucine zipper domain (bZip) (*45*). With the data of photo-isolation chemistry showing lower *Ppargc1a* expression in the Atf3 high group (Fig. 4F) and that *Atf3* induction coincided with the downslope of *Ppargc1a* expression in early FS6KD trajectory analysis (Fig. 3G), we hypothesized that Atf3 could repress *Ppargc1a*, resulting in the repression of the compensatory mechanism in FS6KD mice. The consensus sequence is found in the *Ppargc1a* promoter region in both mouse and human genomes [−144 and −131 from Transcription Start Site (TTS), respectively] (*46*). Therefore, we set up the luciferase reporter assay using the reporter construct containing a 2-kb *Ppargc1a* promoter region. (*46*). In human embryonic kidney (HEK) 293T cells, Atf3 overexpression significantly repressed luciferase signals, which was cancelled by mutant Atf3 lacking the bZip domain. The same effect of Atf3 was replicated in cultured cardiomyocytes (fig. S3, B to D).

These data corroborated our findings from the snRNA-seq trajectory analysis. Although these analyses are candidate approaches and further comprehensive study is required, transient Atf3 induction could account, at least in part, for the repression of the compensatory mechanism in which *Ppargc1a* plays a pivotal part in FS6KD mice, a trigger for disease transition.

### ISR activation follows transient Atf3 induction in FS6KD cardiomyocytes

**Fig. 5.**
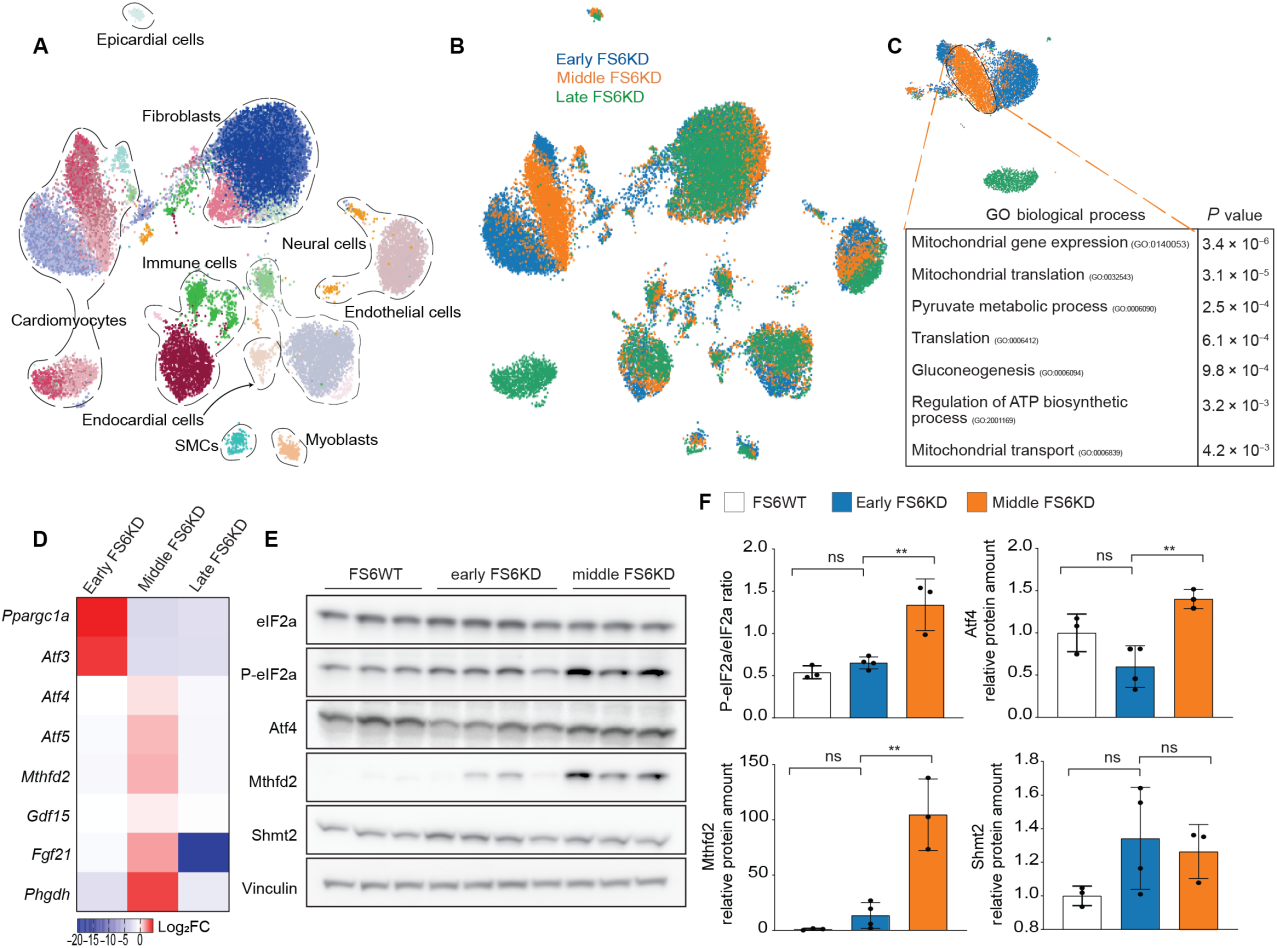
ISR activation follows transient *Atf3* induction in FS6KD cardiomyocytes. (**A** and **B**) UMAP plot of the cardiac cellular populations in the integrated dataset. (A) Colored by Leiden clusters and annotated. (B) Colored by sample identity, early FS6KD (blue), middle FS6KD (orange), and late FS6KD (green). (**C**) UMAP plot of isolated cardiomyocytes colored by sample identity and associated top GO terms of middle FS6KD cardiomyocytes. Fisher exact test was used to calculate *P* values for GO. (**D**) Heatmap displaying expression levels of some *Atf*s and ISR^mt^ genes extracted from DEG list ranked by log_2_FC. (**E** and **F**) Protein analysis of selected ISR-related proteins and enzymes. (E) Immunoblot analysis of eIF2α, phosphorylated eIF2α (p-eIF2α), Atf4, Mthfd2, and Shmt2 in heart lysates from early-stage (*n* = 4 biological replicates) and middle-stage (*n* = 3 biological replicates) mice and (F) quantification of p-eIF2α relative to eIF2α and Atf4, Mthfd2, and Shmt2 relative to WT (*n* = 3 biological replicates) normalized by loading control vinculin. Bars: Means ± SD. Dots: Individual subjects. Ordinary one-way ANOVA and Tukey’s test for multiple comparisons, statistical significance: **P ≤ 0.01. ATP, adenosine triphosphate.

The ATF/CREB TF family has been extensively studied for their roles in ISR^mt^ upon mitochondrial dysfunction, primarily *Atf4* and *Atf5* (*10*, *17*, *18*). In contrast, *Atf3* has received less attention and is generally considered a minor downstream target of *Atf4* or *Atf5* (*16*). However, our investigations revealed no significant up-regulation of *Atf4*, *Atf5*, or other ISR genes in early- or late-stage FS6KD in snRNA-seq analysis compared to WT. This observation led us to evaluate whether ISR^mt^ activation follows *Atf3* up-regulation in the FS6KD heart. To this end, we introduced a 12-week snRNA-seq dataset as the middle stage (Fig. 5, A and B). Cardiomyocytes exhibited the most pronounced changes, and clusters of early- and middle-stage cardiomyocytes were closely connected, implying that the middle-stage dataset represented a state after the transition from the early-stage dataset. The transcriptome of cardiomyocytes at middle stage characterized by GOs included mitochondrial gene expression, mitochondrial translation, and pyruvate metabolism (Fig. 5C). These changes coincided with the up-regulation of ISR response factors, such as *Atf4*, *Atf5*, and *Mthfd2* (Fig. 5D and fig. S4, A to D). Expression of *Ppargc1a* and *Atf3* peaked at early stage and returned to baseline at middle stage, supporting our hypothesis that ISR^mt^ activation followed *Ppargc1a* and *Atf3* down-regulation. To support this further, we incorporated middle-stage cardiomyocytes to early- and late-stage cardiomyocyte trajectory. It demonstrated a connected transition, clearly visible by the overlapping samples in trajectory states (fig. S4, E to G).

To validate our findings obtained from snRNA-seq, we next assessed its protein levels. Western blotting analysis of key molecules in ISR^mt^ using heart lysates from FS6KD mice demonstrated that phosphorylated eIF2α, Atf4, and Mthfd2 in one-carbon metabolism were significantly up-regulated in middle-stage but not in early-stage FS6KD mice (Fig. 5, E and F). We noticed that there is individual variation in the disease transition in female FS6KD. Some mice showed that Atf3 expression was induced much earlier, like 4 weeks. Thus, we defined the disease stages in female FS6KD as follows: early stage (4 to 8 weeks), middle stage (10 to 12 weeks), and late stage (17 weeks). We confirmed Atf3 expression in all the early-stage FS6KD hearts subjected to the Western blotting analysis (Fig. 5, E and F) by immunostaining. We further assessed that immunostaining of Atf4 in FS6WT and FS6KD cardiac tissues showed nuclear localization. Atf4 was expressed in not only cardiomyocytes but also in immune cells and fibroblasts as indicated by snRNA-seq (figs. S1D and S4H). We counted Atf4-expressing nuclei from different time points and observed no significant change in Atf4 levels between WT and FS6KD or FS6KD mice of different disease stages (fig. S4I). These findings exclude the possibility that Atf4 induction in 8-week FS6KD could not be detected by snRNA-seq due to methodological limitation.

Decreased protein levels of mitochondrial translation genes and the subunits and assembly factors of the respiratory complex have been reported during ISR^mt^ (*18**,* *47*). As a result of mitochondrial and cytosolic translation inhibition triggered by phosphorylation of eIF2α, protein levels of the subunits and assembly factors of the respiratory complex are down-regulated. Because the repression of these protein levels is posttranslational, it likely leads to the concomitant up-regulation of RNA expression of these genes by a negative feedback mechanism, which was in line with our data (Fig. 5C and fig. S4J). The protein levels of the subunits of the mitochondrial respiratory complex were down-regulated, although not all. In particular, complex I was severely affected (fig. S4, K to L). We found the down-regulation of *Ppargc1a* at middle stage, which reduces cellular mitochondrial biogenesis. This might have further modulated the above-mentioned negative feedback of gene expression (fig. S4J).

Considering these data, we conclude that ISR^mt^ was activated in the middle stage, not in the early stage, of female FS6KD mice. Atf3 expression precedes ISR^mt^, which is distinctive to the induction of the Atf family found up-regulated during ISR^mt^ activation.

### Genetic ablation of *Atf3* delays heart failure progression in female FS6KD mice

**Fig. 6.**
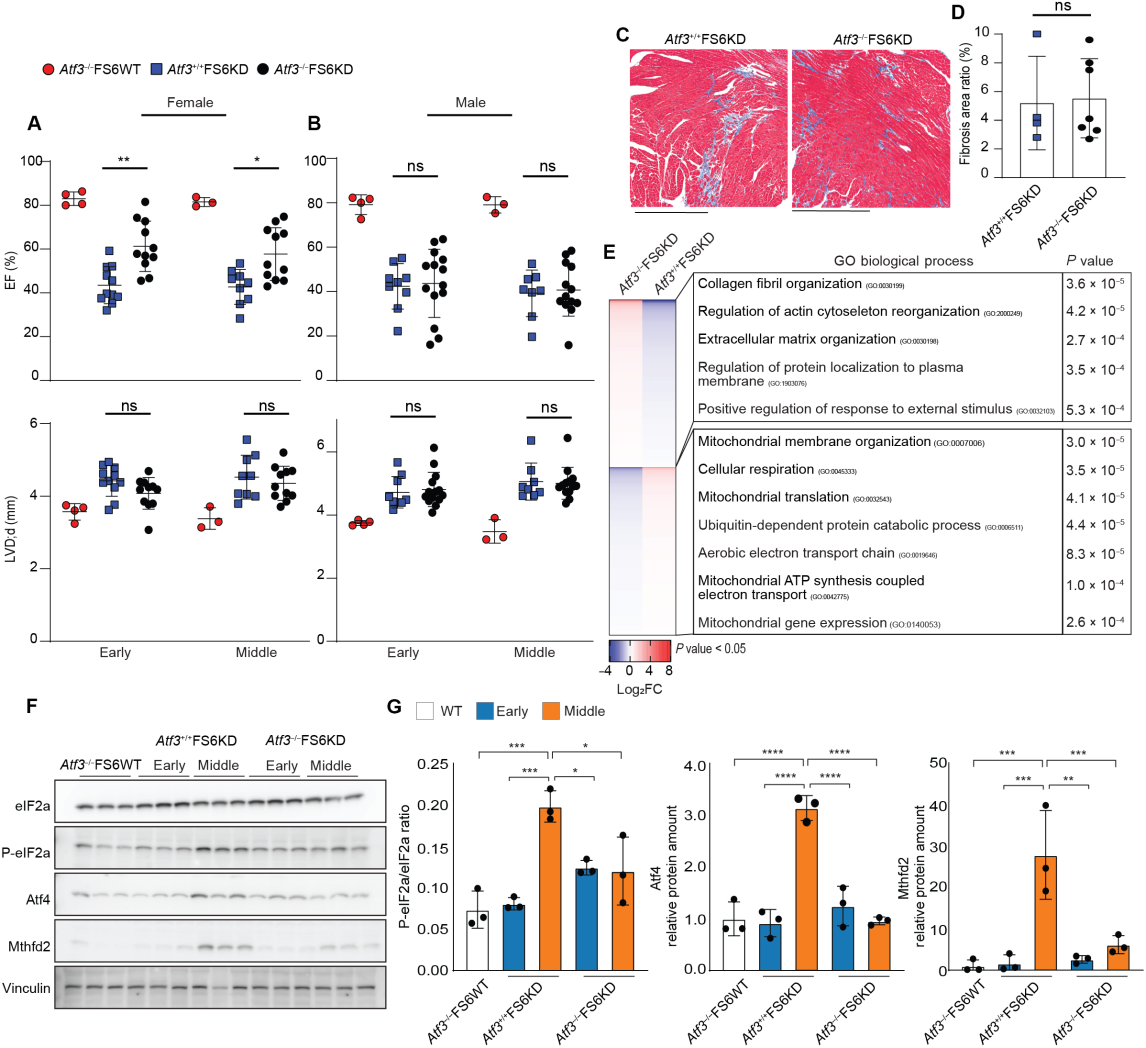
Genetic ablation of *Atf3* delayed the progression of heart failure in female FS6KD mice. (**A** and **B**) Summarized echocardiographic measurements of *Atf3*^−/−^FS6WT, *Atf3*^+/+^FS6KD, and *Atf3*^−/−^FS6KD mice at early and middle stages. (A) EF and LVDd of female mice, early (*n* = 4, 11, and 11 biological replicates) and middle (3, 9, and 11 biological replicates). (B) EF and LVDd of male mice, early (*n* = 4, 9, and 14 biological replicates) and middle (3, 8, and 13 biological replicates). Two-way ANOVA and Tukey’s test for multiple comparisons, (**C**) Masson’s trichrome staining of hearts isolated from middle mice for fibrosis assessment. Field view of *Atf3*^+/+^FS6KD and *Atf3*^−/−^FS6KD. Scale bars, 500 μm. (**D**) Respective quantitative assessment summary of cardiac fibrosis area (*n* = 4 and 7 biological replicates and *n* > 3 technical replicates). Unpaired two-tailed Student’s *t* test. (**E**) Heatmap displaying DEGs extracted from RNA-seq of *Atf3*^+/+^FS6KD or *Atf3*^−/−^FS6KD hearts in middle ranked by log_2_FC and associated top GO terms (*n* = 4 biological replicates). Pairwise two-tailed *t* test was used to calculate *P* values for DEG selection and Fisher exact test to calculate *P* values for GO. (**F** and **G**) Protein analysis of selected ISR-related proteins and enzymes. (F) Immunoblot analysis of eIF2α, p-eIF2α, Atf4, and Mthfd2 in heart lysates from early and middle stages of *Atf3*^+/+^FS6KD and *Atf3*^−/−^FS6KD (*n* = 3 biological replicates each) and (G) quantification of p-eIF2α, Atf4, and Mthfd2 normalized by loading control vinculin. Ordinary one-way ANOVA and Tukey’s test for multiple comparisons. Bars: Means ± SD. Dots: Individual subjects. Statistical significance: *P ≤ 0.05, **P ≤ 0.01, ***P ≤ 0.001, and ****P ≤ 0.0001.

Our preceding findings highlight the role of *Atf3* expression in cardiomyocytes transitioning from metabolic compensation to maladaptation during energy dysfunction. To assess whether the absence of *Atf3* improves cardiac function under OXPHOS dysfunction, we generated *Atf3*^−/−^FS6KD mice via a CRISPR-Cas9–mediated large deletion spanning exon 2 to 4 of *Atf3* in FS6KD fertilized eggs. Echocardiography analysis revealed improvement in EF at 8 weeks and demonstrated sustained stabilization of EF up to 12 weeks in *Atf3*^−/−^FS6KD, with a slight left ventricular diameter increase over time (Fig. 6A). This suggests that *Atf3* is necessary to promote the decline in cardiac contractility observed in FS6KD mice and that *Atf3* knockout delayed heart failure progression in FS6KD mice. This cardioprotective effect disappeared in male counterparts (Fig. 6B). Although we found the improved heart function in female *Atf3*^−/−^FS6KD, there was no significant decrease in fibrotic areas (Fig. 6, C and D). This suggests that cardiac fibrosis in FS6KD is likely not a response to decreased cardiomyocyte contraction but rather a reflection of fibroblast or other cell phenotypes due to mitochondrial dysfunction. *Atf3*^−/−^FS6WT mice displayed a normal cardiac phenotype, consistent with the previous report (*48*), implying that *Atf3* lacks a specific role under normal conditions.

We performed RNA sequencing (RNA-seq) on whole heart tissues to see whether the loss of *Atf3* affects mitochondrial and energy-related processes. RNA-seq revealed that hearts of 12-week *Atf3*^−/−^FS6KD exhibited down-regulation of glycolytic and OXPHOS-dependent metabolic processes, as well as a reduction in mitochondrial maintenance such as translation and transport (Fig. 6E). These GOs were oppositely up-regulated in 12-week FS6KD cardiomyocytes (Fig. 5C), presumably as a feedback mechanism after *Ppargc1a* and *Atf3* down-regulation. This suggests that the loss of *Atf3* prevents cardiomyocytes from transitioning to a compromised state, in which downstream metabolic process genes were up-regulated due to the loss of compensatory mitochondrial biogenesis orchestrated by *Ppargc1a*. We checked the ISR activation by Western blotting using female FS6KD mouse hearts with or without *Atf3* deletion. ISR activation assessed by phosphorylated eIF2a, Mthfd2, was elevated in middle-stage female hearts after *Ppargc1a* was repressed. Also, this ISR activation was inhibited in *Atf3*^−/−^FS6KD (Fig. 6, F and G). These results are in line with the above-mentioned bulk RNA-seq analysis. From these observations, we conclude that *Atf3* plays a pivotal role in disease transitioning in female FS6KD mice and is a possible therapeutic candidate for female MCM.

## DISCUSSION

In this study, spatially resolved transcriptomics and snRNA-seq analysis on the heart of a patient with MCM identified *ATF3* as a high-ranked DEG in the less-damaged cardiomyocyte area, presumably playing a critical role in disease progression. We verified our findings by snRNA-seq analysis of heart samples collected from the MCM mouse model at different time points. Pseudotime trajectory analysis revealed that cardiomyocyte-specific transient up-regulation of *Atf3* preceded ISR^mt^ and functioned as a pivotal trigger for transitioning from a compensatory state to maladaptation, facilitating the onset of female MCM. Last, *Atf3* knockout in FS6KD mice proved a fate-determining role of *Atf3* in MCM progression in a female-specific manner. Our study, therefore, unveils the disease transitioning factor in female MCM preserved across species.

The most remarkable finding is the broad heterogeneity of cardiomyocytes kept in the heart of the patient with MCM. Considering the patient’s severe cardiac failure requiring ventricular assist device implantation, it was conceived that most cardiomyocytes were in the failing stage and quite challenging to trace disease progression. However, the heterogeneous nature of the myocardium of the patient with MCM and spatially resolved transcriptomics enabled us to observe an ongoing transition process within the cardiomyocytes, which further suggests a broader therapeutic intervention window than initially conceived. It should be noted that the transient nature of *Atf3* expression in young mice and heterogeneity of cardiomyocytes from the 8-week FS6KD heart is less complex than the human MCM cardiomyocytes, despite the notable resemblance of cardiomyocyte trajectory observed in the FS6KD hearts. Moreover, human cardiomyocytes displayed an additional trajectory absent in FS6KD mice, emphasizing the intricate heterogeneity of human tissues and the multifaceted stress responses characteristic of patients with MD. This heterogeneity could have stemmed from the RC complex–specific phenotype due to complex IV deficiency, distinct from complex I deficiency in FS6KD mice. Nonetheless, it is conceivable that human MCM progresses slower and less synchronized than the mouse MCM, showing a higher degree of heterogeneity kept in a single time point specimen, which is more suitable for spatially resolved transcriptomics and snRNA-seq analysis to decipher disease progression.

An intriguing finding was the gender-specific protective effect of *Atf3* knockout in FS6KD mice, observed exclusively in female mice and absent in males. We chose female mice for snRNA-seq owing to their slower disease progression, enabling the tracking of early disease-related responses. However, contrary to expectations, this protective response was not observed in male mice. The extent of diseased complex I activity is the same between males and females (fig. S1C), suggesting that the gender-specific protective effect of *Atf3* knockout depends on the downstream response to complex I deficiency. We have not yet understood what molecular mechanism caused the discrepancy in our model, but gender disparities manifest in various diseases, particularly cardiovascular (*49**,* *50*), autoimmune (*51*), Parkinson’s, and Alzheimer’s diseases (*52*). Over the past few decades, studies have also shown gender disparities in mitochondrial function (*53*). In addition, a meta-analysis illustrated consistent gender differences in two domains of mitochondrial biology: higher mitochondrial content in women and elevated reactive oxygen species production in men (*54*). This difference is most likely attributed to sex hormones. For instance, the estrogen-related receptor family is involved in mitochondrial biogenesis and metabolic gene expression (*53*). Estrogen receptors are distributed across mitochondria, nuclei, and plasma membranes, and estrogen’s interaction with mtDNA affects transcription and replication, boosting mitochondrial-related gene expression (*55*). Female mice display enhanced fatty acid utilization and up-regulate genes associated with FAO in muscles during intense exercise to a greater extent than males (*56*). Estrogen treatment enhances mitochondrial biogenesis, adenosine triphosphate production, and *Ppargc1a* expression in hearts after trauma-hemorrhage (*57*). Ppargc1a coactivates estrogen receptor alpha expression (*58*), and its absence increases susceptibility to dilated heart failure in female mice (*59*), underscoring the role of female sex hormones and their regulation of *Ppargc1a* in adapting to compromised metabolism. Therefore, upstream or downstream of *Atf3*-involving transcriptional regulation might be affected by the sex hormones. The difference in gender responsiveness might also be due to other factors involved in the downstream pathways working in parallel or jointly with Atf3 in male FS6KD mice.

Our data revealed that ISR^mt^ expression followed *Atf3* induction. As Atf3 is a member of the Atf family, there is a possibility that *Atf3* expression is an early wave of ISR^mt^ in our model. However, immunohistology analysis and snRNA-seq showed that Atf3 was dominantly expressed in cardiomyocytes. In contrast, Atf4 and Atf5 were expressed in other cell types including fibroblasts and macrophages, suggesting that Atf3 might have a distinct role in the MCM human and mouse model we applied in this study. As another disease model, Kim *et al* (*60*) reported that induction of *Atf3* plays a pivotal role in disease progression in the livers of Zucker diabetic fatty (ZDF) rats, a model of type 2 diabetes exhibiting hepatic steatosis, and patients with nonalcoholic fatty liver disease (*60*). ZDF rats demonstrate the induction of metabolic stress, impaired mitochondrial function, endoplasmic reticulum (ER) stress, and substantial reductions in FAO. Silencing *Atf3* resulted in the re-elevation of *Ppargc1a*, which was down-regulated along with disease progression, thereby restoring FAO levels and mitochondrial function, counterbalancing metabolic dysfunction as well as redox state. Nonetheless, even when ER stress is evident, patients with low ATF3 levels exhibited diminished CHOP expression (*60*), suggesting the potential of ATF3 as a valuable biomarker. As an acute stress response factor, it is not unexpected that *Atf3* induction is evident in various acute injury models (*48**,* *61**–**66*). However, *Atf3* likely plays a distinct role in chronic metabolic dysfunction models, such as MCM in the present study and ZDF rats. Elucidating the upstream factors governing *Atf3* fate determination in MCM, particularly in FS6KD or similar models, necessitates further investigation.

In conclusion, this study reveals that *Atf3* plays a crucial role in orchestrating the shift of cardiomyocytes from compensation to maladaptation. Although further research is essential, these findings offer a previously unidentified insight into how stress responses and adaptations operate in MDs. We believe that spatially resolved transcriptomics and single-cell RNA-seq using patients’ tissues that preserve high heterogeneity will facilitate better understanding and finding therapeutic targets for now intractable MDs, which contain vast individual variations caused by different etiologies and genetic backgrounds.

### Limitations of this study

We demonstrated that *Atf3* up-regulation initiates the shift of cardiomyocytes from a compensated metabolic state to a severe phenotype in a patient with MCM and FS6KD mice. *Atf3* arises as a critical factor regardless of the diseased RC complex, a patient with complex IV deficiency, and FS6KD mice with complex I deficiency, highlighting its importance. However, we analyzed only one MCM patient heart and a single MCM model. It is essential to validate the role of *Atf3* or search for other pivotal factors across diverse MCM or MD manifestations in humans and other mouse models.

## MATERIALS AND METHODS

### Animal models

All the animal experiments were approved by the Institute Animal Care and Use Committee of the National Cerebral and Cardiovascular Center and strictly adhered to the institutional guidelines for animal experiments (approval nos. 20039, 21056, 22078, 23031, and 24009). *Ndufs6*^gt/gt^ heterozygous mouse sperm of mixed genetic background, C57BL/6J and 129/Ola, was provided by D. Thorburn (*25*). The mice were housed in a specific pathogen–free animal facility with a 12-hour light cycle and given a regular chow diet. *Atf3*^−/−^FS6KD mice were created using the CRISPR-Cas9 system as described previously (*67*) by introducing guide RNA/Cas9 (5′-ccagcgcagaggacatccga-3′ for the exon 2 of *Atf3* and 5′-cccagcagccaagagccgtt-3′ for the exon 4 of *Atf3*) protein solution into fertilized eggs of *Ndufs6*^gt/gt^ mice with an electroporator (NEPA21, Nepagene). We obtained F0 founders that had a large deletion in the coding regions of *Atf3* and then intercrossed them to produce functional Atf3-null mice with an FS6KD background and Atf3-null mice without an FS6*^gt^* allele. The genotyping primers used were as follows: 5′-gaggtagg-ctgtcagaccccatgc-3′ for Pr.1, 5′-gcccattctcgggtgcacactatacc-3′ for Pr.2, and 5′-gccacagtggaggatgtggtccc-3′ for Pr.3. All the different genotypes were born in the expected Mendelian ratios and were viable. All experiments and analyses were conducted using female mice unless otherwise stated.

We noticed that there is individual variation in the disease transition in female FS6KD. Some mice showed that Atf3 expression was induced much earlier, like 4 weeks. Thus, we defined the disease stages in female FS6KD as follows: early stage (4 to 8 weeks), middle stage (10 to 12 weeks), and late stage (17 weeks). We confirmed Atf3 expression in all the early-stage FS6KD hearts subjected to the Western blotting analysis by immunostaining.

### Echocardiography

Before echocardiography, mice were anesthetized and maintained with 0.25% isoflurane administered via a mask covering the nose and the mouth of the animals. Transthoracic echocardiography was performed on unconscious mice with a Vevo 3100 imaging system (Visualsonics Inc.). M-mode echocardiographic images were obtained from a short-axis view to measure the left ventricular EF.

### Human MCM heart tissues

The procedure for spatially resolved transcriptomics and snRNA-seq of heart tissues isolated from a patient with MCM was approved by the ethics committee of National Cerebral and Cardiovascular Center (approval no. R20035). Informed consent was obtained from the parents in this study. The cardiac tissues were obtained during ventricular assist device implantation and frozen at −80°C immersed in Bambanker solution (NIPPON Genetics) until analysis. Diagnosis of MCM was based on severe reductions in complex IV assessed by immunohistochemistry (*23*), enzyme activity (*68*), and characteristic mitochondrial features assessed by electron microscopy.

### Spatially resolved transcriptomics

#### Slide preparation

Tissues were obtained during ventricular assist device implantation and were formalin-fixed and then paraffin-embedded (FFPE). RNA quality was performed on FFPE tissue sections (10 μm) using Total RNA Pico assay (Agilent technologies) which were then processed according to the 10X Genomics Visium CytAssist manufacturer’s instructions. Briefly, sections were mounted on a glass slide under ribonuclease (RNase)–free conditions, and libraries were prepared with Visium Human Transcriptome Probe Set v2.0. Sequencing was performed using DNBseq-G400RS (MGI Tech Co.). Raw FASTQ files and histological images were processed using 10X Genomics Space Ranger software (version 2.0.1). Alignment was performed using the GRch38-2020-A reference genome.

#### Data processing

The filtered feature-barcode matrix obtained by Space Ranger was used as input for the Scanpy package (version 1.9.6) in Python (version 3.10.9). Cells with total counts greater than 120,000, counts less than 20,000, and with more than 20% mitochondrial genes were filtered out [scanpy.pp.filter_cells() command]. Genes detected in fewer than 10 cells were excluded. Normalization and log transformation were performed [scanpy.pp.normalize_total() command and sc.pp.log1p() command], and the top 2000 highly variable genes were selected using the “Seurat” algorithm [scanpy.pp.highly_variable_genes() command]. Principal components analysis was conducted [scanpy.pp.pca() command], followed by nearest neighbor analysis [scanpy.pp.neighbors() command], using all default values in Scanpy. Dimensionality reduction was performed using Uniform Manifold Approximation and Projection (UMAP) [scanpy.tl.umap() command], and clustering was carried out using the Leiden algorithm with a resolution of 0.5 [scanpy.tl.leiden() command]. DEGs for each cluster were detected using the Wilcoxon rank-sum test [scanpy.tl.rank_genes_groups() command]. Correlation analysis was performed by Spearman correlation in SciPy (version 1.8.1).

#### Deconvolution

Spatial spots deconvolution was performed using Cell2location (*27*) with hierarchical Bayesian models. Selected hyperparameters are eight cells per spot and 20 detection alpha. Reference dataset was loaded from the human cell atlas database (DOI 10.5281/zenodo.6578046) for healthy human control myocardium (*69*) containing 41,663 nuclei.

### Single-nuclei transcriptomics

#### Nuclei isolation from mouse heart tissues

Hearts of 8- and 17-week female FS6WT, 8-, 12-, and 17-week FS6KD, and 10-week male FS6WT (*n* = 1 for each stage) were minced, and nuclei were isolated according to the Frankenstein protocol (*70*) with minor modifications. All samples, reagents, and steps were kept and performed on ice. Eighty-milligram heart tissue was cut into small pieces in 0.5 ml of ice-cold EZ PREP lysis buffer (Sigma-Aldrich) and homogenized using a 2-ml glass douncer (eight times with pestel A and one time with pestel B) and incubated on ice with additional 1 ml of ice-cold EZ PREP lysis buffer for 3 min with gentle mixing using a wide bore tip two times. Homogenate was filtered through a 70-μm followed by a 20-μm cell strainer (PluriSelect) and centrifuged at 500*g* for 5 min at 4°C to remove cell debris and aggregates. Nuclei pellet was suspended in another 1.5 ml of ice-cold EZ PREP lysis buffer and incubated on ice for 3 min. After centrifugation at 500*g* for 5 min at 4°C, nuclei pellet was incubated with nuclei washing and resuspension buffer consisting of 1× phosphate-buffered saline (PBS), 1% bovine serum albumin (BSA), 0.1% Tween 20, and RNase inhibitor (0.2 U/μl; TAKARA Bio) for 5 min before resuspension with additional 1 ml. Nuclei were centrifuged at 500*g* for 5 min at 4°C, and the nuclei pellet was resuspended with 0.5 ml of nuclei washing and resuspension buffer containing 4′,6-diamidino-2-phenylindole (DAPI) stain (DOJINDO) for nuclei labeling. Final nuclei count was determined by counting nuclei with a hemocytometer (NanoEntek) by fluorescent microscopy (Keyence, BZ-X810).

#### FACS sorting

Isolated nuclei, kept on ice, were immediately sorted by fluorescence-activated cell sorting (FACS) using a FACSAria Fusion cell sorter (Becton Dickinson, National Cerebral and Cardiovascular Center). The 355-nm ultraviolet laser was used for analysis and nuclei sorting, while scattering detection of the 488-nm blue laser was used to record forward-scatter characteristics and side-scatter characteristics. Data were recorded and analyzed for gating and single-nuclei selection using BD FACSDiva (ver. 8.0.3) software. Final samples were centrifuged at 500*g* for 10 min at 4°C, and pellets were resuspended with nuclei washing and resuspension buffer not containing Tween 20, to a final concentration of 1000 nuclei/μl. The final nuclei count was determined by counting nuclei with a hemocytometer (NanoEntek) using an all-in-one microscope (Keyence, BZ-X810).

#### Nuclei isolation from human heart tissues

Nuclei were isolated using a Singulator 100 system (S2 Genomics Inc.) according to the manufacturer’s recommendations. All samples, reagents, and steps were kept and performed on ice. Small 11-mg heart tissue was cut into small pieces and inserted into nuclei cartridge (S2 Genomics Inc.) with an RNase inhibitor (0.2 U/μl). Default protocol 2a was selected with one disruption cycle and 5 min incubation posthomogenization in 4 ml of nuclei isolation reagent (S2 Genomics Inc). Sample was passed through 40 μm in the cartridge, followed by a rinse with nuclei storage reagent (S2 Genomics Inc). Collected nuclei were centrifuged at 500*g* for 10 min at 4°C, and pellets were resuspended with 4 ml of nuclei washing and resuspension buffer containing 1× PBS, 1% BSA, and RNase inhibitor (0.2 U/μl). Additional centrifugation at 500*g* for 5 min at 4°C was performed, and the nuclei pellet was resuspended with washing and resuspension buffer containing DAPI stain (DOJINDO) for nuclei labeling. The final nuclei count was determined by counting nuclei with a hemocytometer (NanoEntek) by fluorescent microscopy (Keyence, BZ-X810).

#### Library preparation, sequencing, and read processing

Droplets capturing single nucleus from whole heart nuclei suspension were used for library generation in the 10X Genomics Chromium controller according to the manufacturer’s instructions in the Chromium Single Cell 3′ Reagent Kit v.3 User Guide. Additional components used for library preparation include the Chromium Single Cell 3′ Library and Gel Bead Kit v.3 (PN-1000092). Libraries were prepared according to the manufacturer’s instructions using the Chromium Single Cell 3′ Library, the Gel Bead Kit v.3 (PN-1000092), and the Chromium i7 Multiplex Kit (PN-120262). Final libraries were sequenced on Illumina NovaSeq6000. All libraries were sequenced to a depth of at least 20,000 total mean reads per nucleus. The Cell Ranger (ver. 5.0.0 for mouse and ver. 6.0.0 for human) pipeline provided by 10X Genomics was used to process raw sequencing reads. Reads were aligned to mouse mm10 and to human GRCh38 references with pre-mRNA intronic regions. Gene-barcode matrices were created by quantifying Unique Molecular Identifiers (UMI) counts per gene per cell, and the data were aggregated and normalized creating a combined gene-barcode matrix. Individual matrices of samples were used for downstream processing and analysis.

#### Mouse sample integration, gene matrix preprocessing, filtering, and cell clustering

Scanpy (*71*) (ver. 1.9.6) package was used for further preprocessing, filtering, and clustering of filtered gene matrices obtained by Cell Ranger. For comparison between two or more datasets, the ComBat (*72*) algorithm was used for batch effect correction. Data matrices were first converted to anndata objects where each dataset was annotated under a new observation column “sample,” followed by merging (concatenate command). Poor quality cells were removed by filtering out cells [scanpy.pp.filter_cells() command] based on the number of counts and mitochondrial gene percentage. Cells with fewer than 200 genes and with higher than 0.5% mitochondrial genes were excluded. Genes expressed in less than three cells were considered outliers and were excluded by filtering in the preprocessing step. To allow counts to become comparable among all cells, library size was corrected by normalizing [scanpy.pp.normalize_total() command] to a scale factor of 10,000, followed by log transformation [sc.pp.log1p() command] and annotation of highly variable genes [scanpy.pp.highly_variable_genes() command] with n_top_genes set to None. Scaling of the data to follow a linear regression model [scanpy.pp.scale() command] was performed with max_value of 10. Dimensionality reduction was computed using linear principal components analysis [scanpy.tl.pca() command], and a number of significant principal components (PC) to be used for clustering were determined by plotting each PC’s variance ratio. Next, selected PCs were used to calculate the neighborhood graph [scanpy.pp.neighbors() command] that is automatically embedded to compute the UMAP (*73*) graph [scanpy.tl.umap() command] for two dimensional visualization of cells. Communities of cells were detected by the Leiden (*74*) algorithm [scanpy.tl.leiden() command] with the default resolution of 1. The ComBat algorithm was then used [scanpy.pp.combat() command] to remove batch variation between samples, and significant PCs were selected to calculate the neighborhood graph and UMAP topology.

#### Human sample gene matrix preprocessing, filtering, and cell clustering

Doublets in human sample matrix were high due to not using FACS. Doublets were removed from raw count matrix before preprocessing by Scrublet (*75*) (ver. 0.2.3) Python package with upstream data log transformation. The Scrublet object was initialized [scrublet.scrublet() command] with a sparse raw count matrix and an expected doublet rate of 0.076. Scores were calculated, and binary prediction was assigned for each cell [scrublet.scrub_doublets() command] with 30 PCs and log_transform set to True. Cells predicted as True doublets were removed from the count matrix. Scanpy was used for further preprocessing, filtering, and clustering of doublet-free matrix. Analysis was performed as described for a mouse without batch correction and with few modifications. Cells with fewer than 500 genes and genes expressed in less than ten cells were considered outliers and were excluded by filtering. Annotation of highly variable genes [scanpy.pp.highly_variable_genes() command] with n_top_genes is set to 2000 and with flavor set to Seurat_3. To completely exclude doublets before cardiomyocytes subclustering, cells with high expression of one other cell marker gene were removed, based on expression levels and frequency, and we removed cells expressing *VWF*. For the integration with a healthy sample, a single-nuclei Robj.gz file of 11-year donor sample (*36*) was downloaded from the human cell atlas database [Gene Expression Omnibus (GEO) accession number: GSE183852]. Robj file was converted from R object to h5ad file in R (ver. 4.1.0) using sceasy (*76*) (ver. 0.0.6) library (sceasy::convertFormt command) and then used in Scanpy for downstream integration. Batch Balanced k Nearest Neighbors (BBKNN) (*77*) (ver. 1.5.1) integration was then performed on nonscaled, log-transformed, and normalized concatenated anndata object (scanpy.external.pp.bbknn command) using sample as the batch key including 30 PCs. UMAP was lastly run on the integrated space (scanpy.tl.umap command).

#### Differential gene expression analysis and annotations

To identify highly expressed genes in Leiden clusters, DEGs were ranked [scanpy.tl.rank_genes_groups() command] using the Wilcoxon rank-sum test. Genes were ranked by log_2_ fold change (log_2_FC) values and *P* values. Top statistically significant genes were used for cell type manual annotation based on the expression of cell-specific marker genes manually curated from literature.

#### Subclustering

Cardiomyocytes were isolated and reclustered, the UMAP graph was computed, and the Leiden algorithm was used with a resolution of 1. For the sake of reproducibility, subclustered cardiomyocytes were stored as anndata file and further analyzed. DEG analysis between different samples was then performed using the Wilcoxon rank-sum test, and genes were ranked by log_2_FC values and *P* values. Ranked gene lists were used for comparative analysis and downstream GO analysis.

#### Pseudo-time trajectory analysis

Integrated reclustered cardiomyocytes were used for trajectory analysis. The ForceAtlas2 (FA2) (*78*) (ver. 0.3.5) algorithm was used to draw a single-cell graph [scanpy.tl.draw_graph() command] using the anndata object previously calculated PCA space and the neighborhood graph. To denoise the graph, diffusion maps were computed [scanpy.tl.diffmap() command] using the first 15 components, and neighborhood distances were calculated between diffusion components [scanpy.pp.neighbors() command] to draw graph on the diffusion map [scanpy.tl.draw_graph() command]. Clusters were detected by the Leiden algorithm [scanpy.tl.leiden() command] with the default resolution of 1. Next, the partition-based graph abstraction (PAGA) (*29*) (ver. 1.2) graph was calculated [scanpy.tl.paga() command] based on embedding connectivity maps of UMAP clusters calculated by the Leiden algorithm. The single-cell graph was recomputed [scanpy.pl.draw_graph() command] on the basis of the PAGA graph calculated earlier. To assign a root for pseudotime temporal path tracing, the first cluster with the highest *Ppargc1a* expression was chosen.

#### GO analysis

Gene lists were filtered with a *P* value cutoff of <0.01 and a log_2_FC value of >1.5. GO analysis was performed on gene lists using EnrichR (*79**–**81*), and *P* values were calculated with the Fisher exact test.

### Immunohistology staining

Freshly dissected heart tissues were fixed in ice-cold 4% paraformaldehyde phosphate buffer solution (WAKO) for 24 hours followed by serial immersion of the tissue in 10, 20, and 30% sucrose in PBS for 24 hours each. Tissues were embedded in optimal cutting temperature (OCT) compound (Sakura Finetek) and frozen by chilling at −80°C. For Atf3 staining antigen retrieval, sections (8 μm) were submerged in 1× citrate buffer (pH 6.0) (Abcam) and microwaved for 2 min followed by 10-min boiling in 98°C water bath. Sections cooled for at least 30 min at room temperature were submerged under running hot water for 1 min and washed with 1× buffer two times for 3 min and then 1× PBS three times for 3 min. For Atf4 staining antigen retrieval, sections (8 μm) were submerged in 1× citrate buffer, (pH 6.0) (Abcam) and autoclaved for 10 min at 120°C. Sections were cooled in closed autoclave until temperature reached 60°C. Following cooling, tissues were submerged under running hot water for 1 min and washed three times with 1× PBS for 3 min. Sections were blocked with Blocking One Histo (Nacalai Tesque) for 10 min at room temperature and then incubated with primary antibodies at 4°C overnight (anti-Atf3; 1:500; Abcam, catalog no. ab254268; anti-Atf4; 1:100; Abcam, catalog no. ab31390). For double staining, 4°C overnight incubation was followed by three times PBS washing and then incubation with goat anti-cTnnI3 (1:500; Abcam catalog no. ab56357) for an additional overnight at 4°C. Three times washing with PBS was followed by secondary antibodies goat anti-rabbit immunoglobulin G (IgG) Alexa Fluor 555 (Abcam, catalog no. ab150078), donkey anti-goat IgG Alexa Fluor 488 (Thermo Fisher Scientific, catalog no. A-11055), and Hoechst incubation for 1 hour at room temperature. Sections were washed and mounted with VECTASHIELD (Vector Laboratories). Images were obtained using a confocal laser scanning microscope (Olympus, FV3000).

### Nuclei counting

Atf3- and Atf4-positive nuclei were counted using the open-source software QuPath (*82*) v.0.5.0. Three or more areas at ×20 magnification were randomly selected. Macro was created by training an object classifier on several random images by classifying nuclei into positive and negative. Classifier was then applied on all images after cellular detection was applied, and all nuclei were selected per area.

### Photo-isolation chemistry

Spatial transcriptomics of Atf3-positive cells in mouse was performed using photo-isolation chemistry as described (*43*, *44*). Briefly, fresh-frozen tissue sections embedded in OCT compound (Sakura Finetek) after ice-cold PBS perfusion and frozen by incubating on isopentane chilled in liquid nitrogen were permeabilized with HCl (Nacalai Tesque), followed by in situ mRNA reverse transcription using photo-caged oligodeoxynucleotides (ODNs) primers (Glen Research). After first-strand synthesis, anti-Atf3 antibody was used to identify the region of interest by immunostaining. Tissue sections were divided into separate groups; high Atf3 and low Atf3 sections. Ultraviolet irradiation (light-emitting diode light, Prizmatix) was used for uncaging ODNs allowing in vitro transcription reaction. Libraries were further reverse-transcribed and paired-end sequenced on NovaSeq6000 (Illumina). Final reads were mapped to reference genome (GRCm38) using HISAT2 (*83*) (ver. 2.1.0). Analysis was performed on the web-based integrated Differential Expression and Pathway (iDEP) (*84*) (ver. 1.1) Analysis tool. For DEG selection, a two-tailed *t* test was performed on normalized processed counts extracted from iDEP, followed by log_2_FC calculations. Gene lists were filtered with a *P* value cutoff of <0.05. GO analysis was performed on gene lists using EnrichR, and *P* values were calculated with the Fisher exact test.

### Western blotting

Western blot analysis was performed based on (*18*) with minor modifications (*18*). Snap-frozen mouse heart tissues collected from FS6WT, early and middle FS6KD, Atf3^−/−^FS6WT, early and middle Atf3^+/+^FS6KD, and early and middle Atf3^−/−^FS6KD were homogenized with TissueLyser LT (QIAGEN) at 50 oscillations for 3 min in lysis buffer consisting of 50 mM tris-HCl (pH 7.5), 150 mM NaCl, 5 mM MgCl_2_, 1 mM dithiothreitol, 10% glycerol, 2% SDS, 1% Triton X-100, protease inhibitor cocktail (Roche), and phosphatase inhibitor cocktail (PhosSTOP, Roche). Twenty micrograms of total protein was loaded for SDS–polyacrylamide gel electrophoresis, followed by transfer to nitrocellulose membranes. Immunoblotting was performed with the following antibodies: eIF2α [1:2000; Cell Signaling Technology (CST), catalog no. 5324], P-eIF2α (1:1000; CST, catalog no. 3597), Mthfd2 [1:4000; Proteintech Group, Inc. (proteintech) (PGI), catalog no.12270-1-AP], Shmt2 (1:1000; Abcam, catalog no. ab180786), Atf4 (1:1000; Abcam, catalog no. ab216839), Cox6a1 (1:2000; Abcam, catalog no. ab110265), Ndufs3 (1:1000; Abcam, catalog no. ab195807), Ndufs4 (1:4000; Abcam, catalog no. ab137064), Nduf8b (1:1000; Abcam, catalog no. ab180786), and Atp5f1 (1:1000; Abcam, catalog no. ab117991). Protein amounts were quantified using GelAnalyzer software (GelAnalyzer v23.1.1 by I. Lazar Jr. and I. Lazar Sr.) and normalized against vinculin (1:1000; CST, catalog no. 4650). For band detection, detection parameters were all set to 1 for height, slope, break threshold, and smoothing. Baseline for peaks was detected using the rolling ball method with a peak tolerance of 10 set for all bands.

### RNA sequencing

RNA was isolated from 12-week hearts of *Atf3*^−/−^FS6KD or *Atf3*^+/+^FS6KD mice using an RNeasy fibrous kit (QIAGEN). Libraries were prepared for sequencing using a TruSeq Stranded mRNA sample prep kit (Illumina) according to the manufacturer’s instructions. Whole transcriptome sequencing was performed using a NovaSeq6000 platform (Illumina) in a 101-bp single-end mode. Sequenced reads were mapped to the mouse reference genome sequence (GRCm38_p6). BAM files were then each converted into one integrated count file using FeatureCounts (ver. 2.0.0). The read count file was normalized by the CPM (counts per million) method using iDEP. Genes expressed with >1 CPM in at least one of the samples of any condition were only used for the analysis. For DEG selection, two-tailed *t* test was performed on normalized processed counts extracted from iDEP, followed by log_2_FC calculations. Gene lists were filtered with a *P* value cutoff of <0.05. GO analysis was performed on gene lists using EnrichR, and *P* values were calculated with the Fisher exact test.

### Mitochondrial respiratory complex enzymology analysis

Mouse tissues were homogenized, and assays were performed on postnuclear supernatants as described previously (*85*). Activity of complexes I to IV in the mouse heart, was expressed relative to citrate synthase activity.

### Luciferase reporter assay

HEK293T cells or rat neonatal cardiomyocytes were cotransfected with expression plasmids for *Atf3*, *Atf3* mutant lacking bZip domain (amino acid position: 86 to 149) in pEF-DEST51 vector (Invitrogen), or *lacz* expressing plasmid, pEF/GW-51/lacz (Invitrogen), together with a reporter gene plasmid containing 2 kb of mouse *Ppargc1a* promoter or without promoter, pGL3-Basic by using PEI max for HEK293T cells, ViaFect (Promega) for cardiomyocytes, respectively. The reporter plasmid containing 2 kb of mouse *Ppargc1a* promoter was obtained from Addgene (#8887) (*46*). After 48 hours, cells were harvested, and luciferase activity was determined. Expression of reporter genes was normalized to the *lacz* expressing group. Luciferase activity was assayed using the luciferase assay system (E1500; Promega) according to the manufacturer’s instructions and was measured using a POLARstar Omega6 microplate luminometer (BMG Labtech)

### Statistical analysis

Data were analyzed using GraphPad Prism 8 and were considered significant when *P* < 0.05. Data between two groups were compared with the Student’s *t* test. Data from more than two groups were evaluated with ordinary one-way analysis of variance (ANOVA) followed by Tukey’s post hoc analysis, and data from compound two or more groups were evaluated with two-way ANOVA followed by Tukey’s post hoc analysis. All data are presented as means ± SD. Schematic diagrams and images were created and processed by Adobe Illustrator (ver. 27.0.1).
