# Supplementary material for: Atf3 controls transitioning in female mitochondrial cardiomyopathy as identified by spatial and single-cell transcriptomics
Source: Sci Adv. 2025 Apr 4;11(14):eadq1575. doi: 10.1126/sciadv.adq1575 (PMC11970478; doi:10.1126/sciadv.adq1575)
Supplement: Supplementary file 1 — Figs. S1 to S4 [file sciadv.adq1575_sm.pdf]

Science Advances
AAAS

Supplementary Materials for

Atf3 controls transitioning in female mitochondrial cardiomyopathy as identified by spatial and single-cell transcriptomics

Tasneem Qaqorh *et al.*

Corresponding author: Yasunori Shintani, shintani.yasunori@ncvc.go.jp

Sci. Adv. **11**, eadq1575 (2025)
DOI: 10.1126/sciadv.adq1575

This PDF file includes:

Figs. S1 to S4

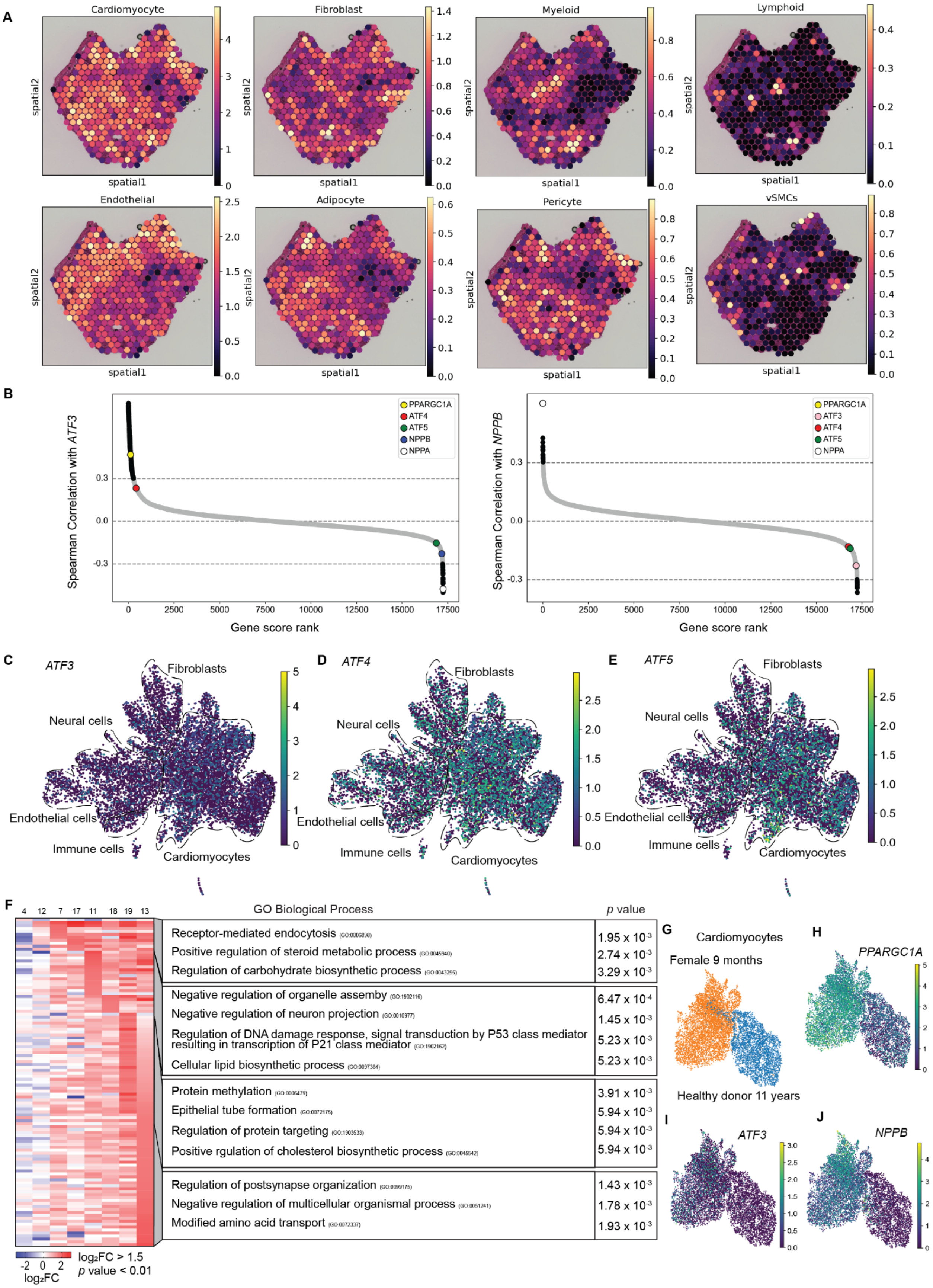
A
Cardiomyocyte
Fibroblast
Myeloid
Lymphoid
Endothelial
Adipocyte
Pericyte
vSMCs
spatial1
spatial2
B
Spearman Correlation with ATF3
Spearman Correlation with NPPB
Gene score rank
PPARGC1A
ATF3
ATF4
ATF5
NPPB
NPPA
C
ATF3
D
ATF4
E
ATF5
Fibroblasts
Neural cells
Endothelial cells
Immune cells
Cardiomyocytes
F
4 12 7 17 11 18 19 13
GO Biological Process
p value
Receptor-mediated endocytosis (GO:0006898)
1.95 x 10-3
Positive regulation of steroid metabolic process (GO:0045940)
2.74 x 10-3
Regulation of carbohydrate biosynthetic process (GO:0043255)
3.29 x 10-3
Negative regulation of organelle assemby (GO:1902116)
6.47 x 10-4
Negative regulation of neuron projection (GO:0010977)
1.45 x 10-3
Regulation of DNA damage response, signal transduction by P53 class mediator resulting in transcription of P21 class mediator (GO:1902162)
5.23 x 10-3
Cellular lipid biosynthetic process (GO:0097384)
5.23 x 10-3
Protein methylation (GO:0006479)
3.91 x 10-3
Epithelial tube formation (GO:0072175)
5.94 x 10-3
Regulation of protein targeting (GO:1903533)
5.94 x 10-3
Positive rgulation of cholesterol biosynthetic process (GO:0045542)
5.94 x 10-3
Regulation of postsynapse organization (GO:0099175)
1.43 x 10-3
Negative regulation of multicellular organismal process (GO:0051241)
1.78 x 10-3
Modified amino acid transport (GO:0072337)
1.93 x 10-3
log2FC
log2FC > 1.5
p value < 0.01
G
Cardiomyocytes
Female 9 months
Healthy donor 11 years
H
PPARGC1A
I
ATF3
J
NPPB

Fig. S1. ***ATF3* upregulation is distinct from *ATF4* and *ATF5***

(**A**) Abundance of different cellular groups in each deconvolved spatial transcriptomics spot distributed across the myocardium region of female MCM patient.

(**B**) Per-gene score Spearman correlation with *ATF3* and *NPPB*. Colored dots show correlation score for each annotated gene. Correlation magnitude cutoff was annotated as 0.3 for positive correlation.

(**C-E**) Expression shown as cardiac cellular population UMAP features of (**C**) *ATF3*, (**D**) *ATF4*, and (**E**) *ATF5*.

(**F**) Heatmap displaying top upregulated genes in the cellular states of the second trajectory selected cellular states ranked by $\log_2$FC and associated top GO terms. Fisher exact test was used to calculate *p*-values for GO.

(**G**) UMAP plot of ventricular cardiomyocytes from 11-year-old female donor dataset (blue) integrated with cardiomyocytes from the MD patient (orange). Expression shown as cardiomyocytes UMAP features of (**H**) *PPARGC1A*, (**I**) *ATF3*, and (**J**) *NPPB*.

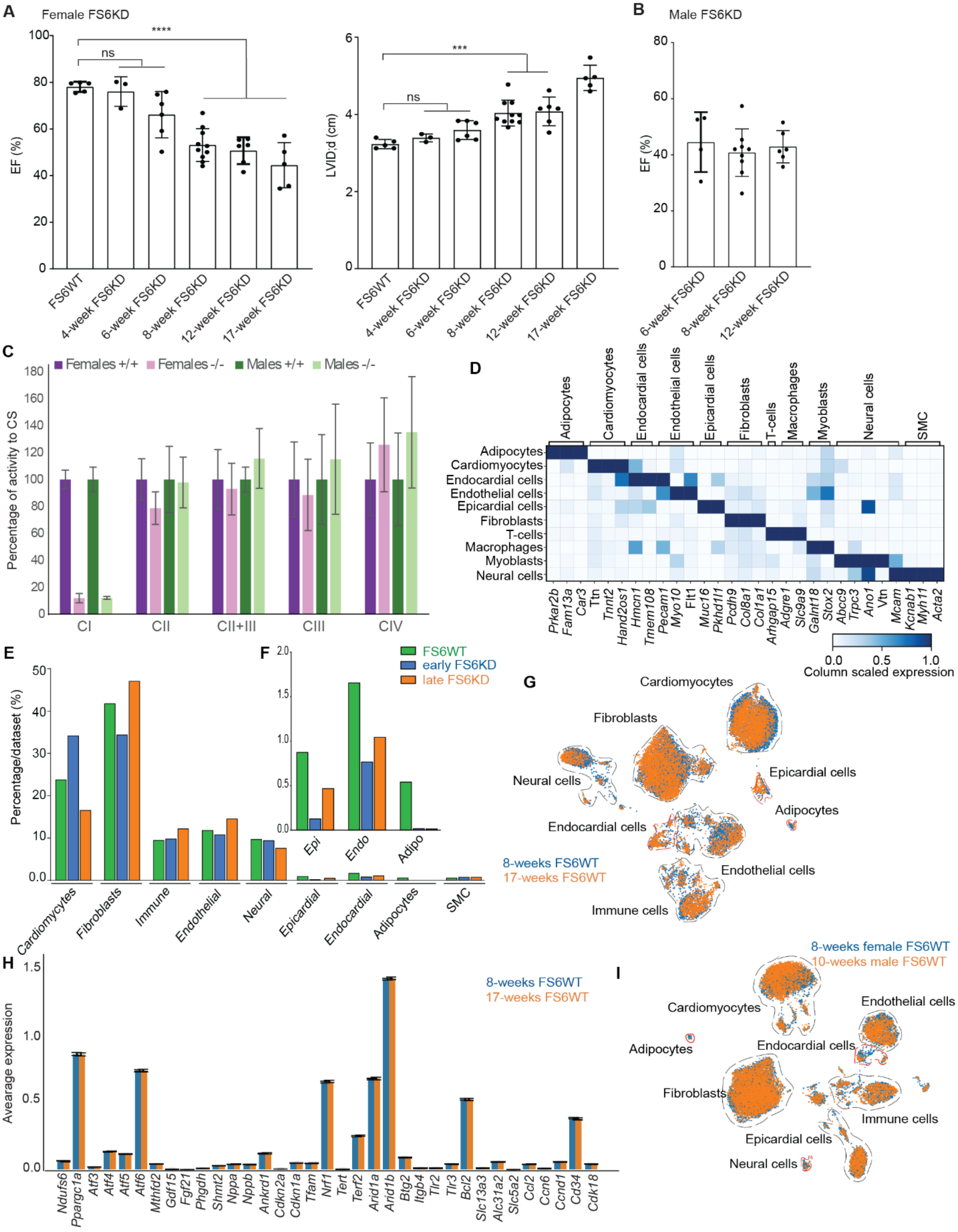

A
Female FS6KD
****
ns
EF (%)
FS6WT
4-week FS6KD
6-week FS6KD
8-week FS6KD
12-week FS6KD
17-week FS6KD
***
ns
LVID;d (cm)
B
Male FS6KD
EF (%)
6-week FS6KD
8-week FS6KD
12-week FS6KD
C
Females +/+
Females -/-
Males +/+
Males -/-
Percentage of activity to CS
CI
CII
CII+III
CIII
CIV
D
Adipocytes
Cardiomyocytes
Endocardial cells
Endothelial cells
Epicardial cells
Fibroblasts
T-cells
Macrophages
Myoblasts
Neural cells
SMC
Prkar2b
Fam13a
Car3
Ttn
Tnnt2
Hand2os1
Hmcn1
Tmem108
Pecam1
Myo10
Flt1
Muc16
Pkhd1l1
Pcdh9
Col8a1
Col1a1
Arhgap15
Slc9a9
Adgre1
Galnt18
Stox2
Abcc9
Trpc3
Ano1
Vtn
Mcam
Kcnab1
Myh11
Acta2
Column scaled expression
E
Percentage/dataset (%)
Cardiomycytes
Fibroblasts
Immune
Endothelial
Neural
Epicardial
Endocardial
Adipocytes
SMC
F
FS6WT
early FS6KD
late FS6KD
Epi
Endo
Adipo
G
Cardiomyocytes
Fibroblasts
Neural cells
Epicardial cells
Adipocytes
Endocardial cells
8-weeks FS6WT
17-weeks FS6WT
Endothelial cells
Immune cells
H
Avearage expression
8-weeks FS6WT
17-weeks FS6WT
Ndufs6
Ppargc1a
Atf3
Atf4
Atf5
Atf6
Mthfd2
Gdf15
Fgf21
Phgdh
Shmt2
Nppa
Nppb
Ankrd1
Cdkn2a
Cdkn1a
Tfam
Nrf1
Tert
Terf2
Arid1a
Arid1b
Btg2
Itgb4
Tlr2
Tlr3
Bcl2
Slc13a3
Alc31a2
Slc5a2
Ccl2
Ccn6
Ccnd1
Cd34
Cdk18
I
8-weeks female FS6WT
10-weeks male FS6WT
Cardiomyocytes
Endothelial cells
Adipocytes
Endocardial cells
Fibroblasts
Immune cells
Epicardial cells
Neural cells

Fig. S2. Female MCM mice demonstrated progressing phenotype and different cellular composition.

(**A** and **B**) Serial EF measurements of (**A**) female FS6KD in comparison to 8-week FS6WT mice (n=5, 3, 6, 10, 6, 5 biological replicates) and (**B**) male FS6KD mice starting 6 weeks (n=4, 9, 6 biological replicates). Bars: mean ± SD. Dots: individual subjects. Ordinary one-way ANOVA and Tukey's test for multiple comparisons, statistical significance: ***$p \leq 0.001$, ns; not significant.

(**C**) Comparison of enzymatic activity of mitochondrial respiratory complexes I-IV (CI, CII, CII+III, CIII, CIV) between male and female FS6KD hearts. Activity of respiratory complexes expressed relative to citrate synthase (CS). N=3, 4, 6, 8, for Female +/+, Female -/-, Male +/+, Male -/-, respectively. +/+; FS6WT, -/-; FS6KD.

(**D**) Heatmap displaying expression of different cardiac marker genes generated from the integrated dataset and their cell type annotations.

(**E** and **F**) Percentages of cellular populations per sample within the integrated dataset with (**E**) focus on percentage of epicardial cells, endocardial cells, and adipocytes.

(**G** and **H**) UMAP plot of cardiac cellular populations in the integrated dataset, colored by sample identity and annotated, 8-week FS6WT (Blue) and 17-week FS6WT (Orange), and the (**H**) average expression of selected genes per dataset. UMAP plot of cardiac cellular populations in the integrated dataset, colored by sample identity and annotated, 8-week FS6WT (Blue) and 17-week FS6WT (Orange).

(**I**) UMAP plot of cardiac cellular populations in the integrated dataset, colored by sample identity and annotated, 8-week female FS6WT (Blue) and 10-week male FS6WT (Orange).

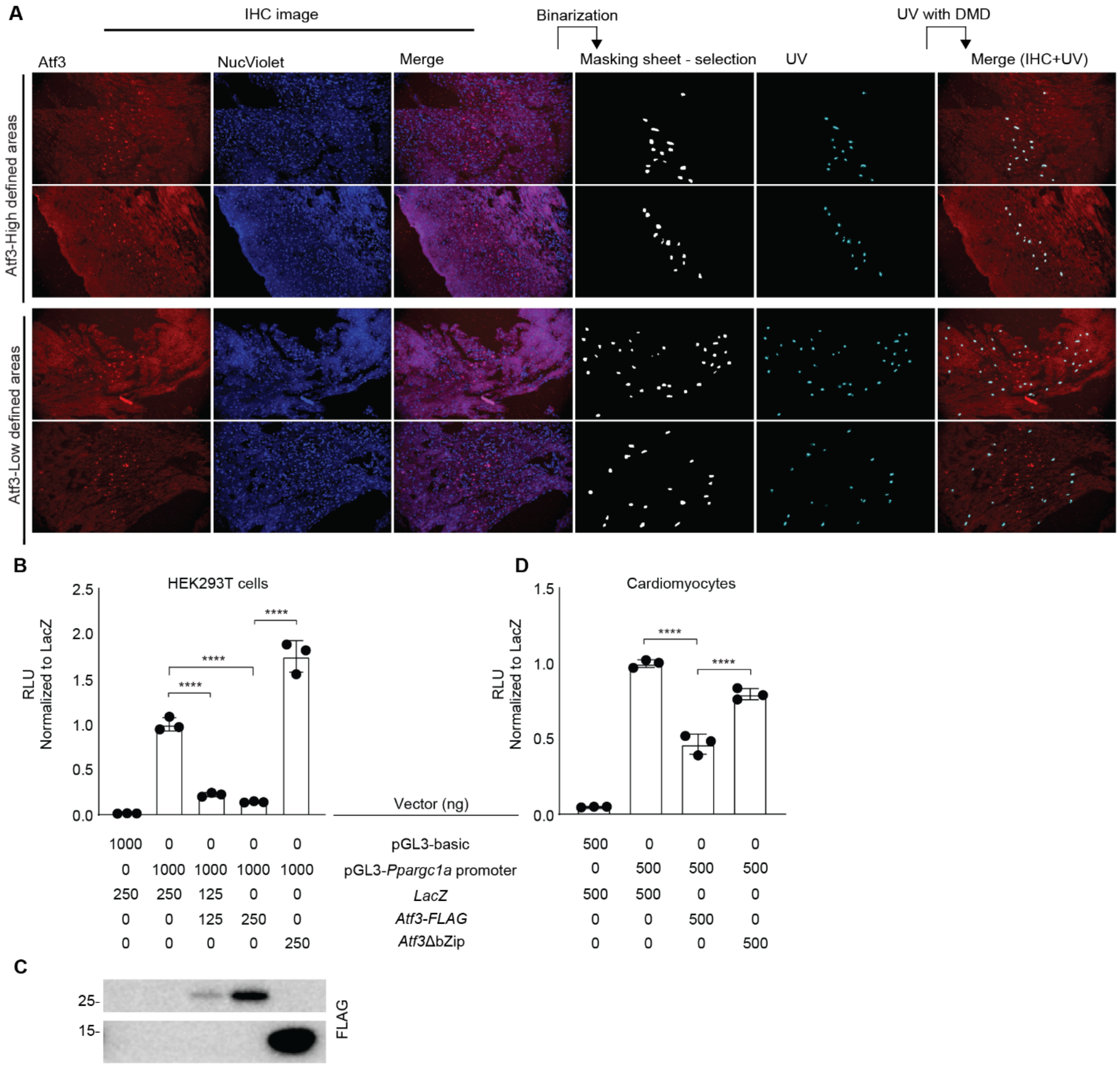

Fig. S3. Spatially resolved transcriptomics of Atf3-expressing clusters by PIC.

(**A**) Atf3 immunostaining (red) and nuclei staining (blue) for the selection of Atf3 positive nuclei. Masking sheets paired with IHC and UV irradiation are shown. Representative images were shown for Atf3-High and Atf3-low defined areas (n=3 biological replicates and n>3 technical replicates per group).

(**B-D**) Luciferase reporter assay using overexpressed 2kb *Ppargc1a* promoter (pGL3-*Ppargc1a* promoter) in (B) HEK293T cells with or without Atf3 (*Atf3*-FLAG) overexpression, confirmed by (C) immunoblotting of FLAG from cell lysate, and rescued by truncated Atf3 lacking bZip binding domain (*Atf3*ΔbZip). Repression of *Ppargc1a* promoter was replicated in (D) cardiomyocytes and rescued by Atf3ΔbZip. Bars: mean ± SD. Dots:

individual subjects. Ordinary one-way ANOVA and Tukey's test for multiple comparisons, statistical significance: ****$p \leq 0.0001$

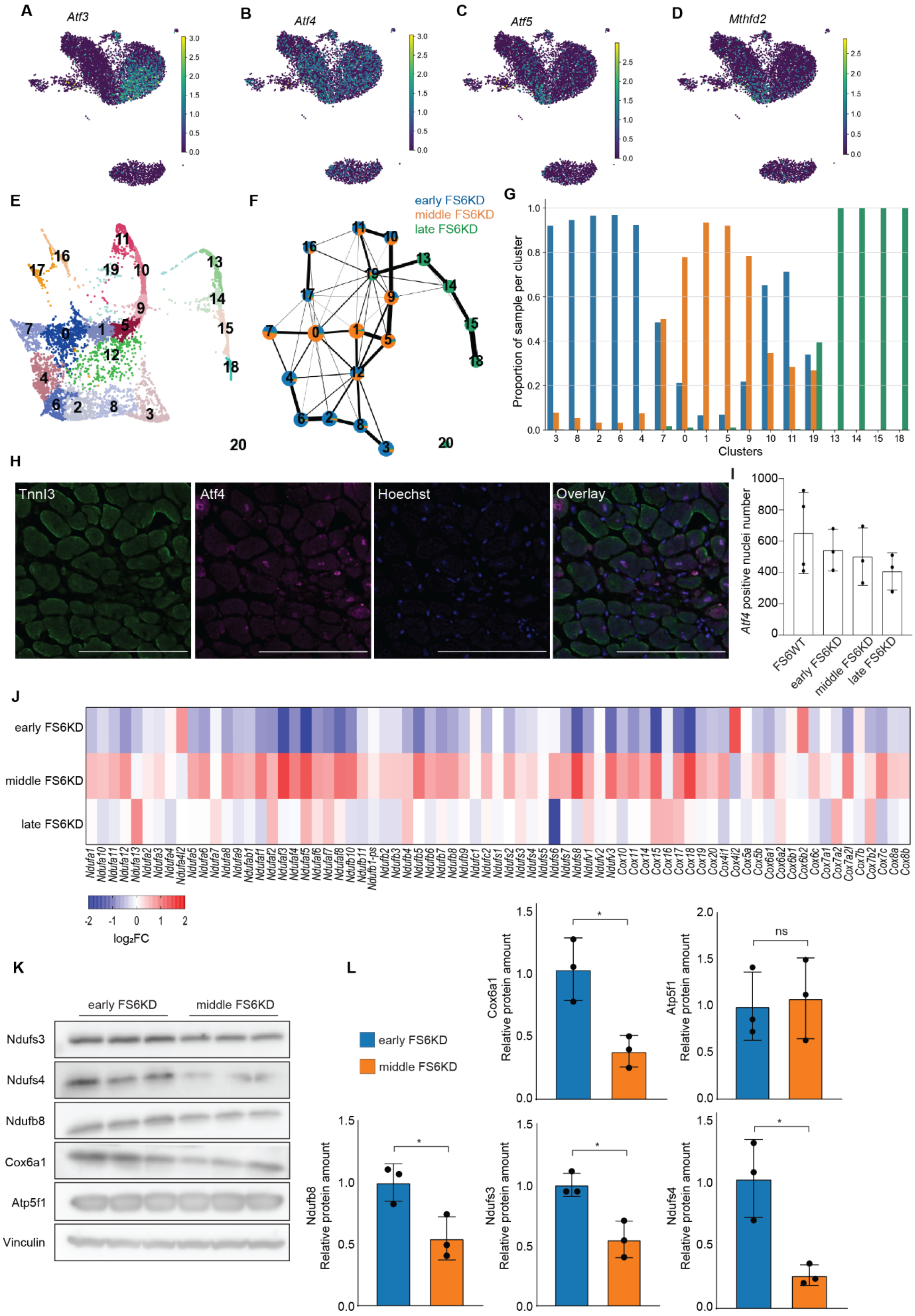

A
Atf3
B
Atf4
C
Atf5
D
Mthfd2
E
F
early FS6KD
middle FS6KD
late FS6KD
G
Proportion of sample per cluster
Clusters
H
Tnnl3
Atf4
Hoechst
Overlay
I
Atf4 positive nuclei number
FS6WT
early FS6KD
middle FS6KD
late FS6KD
J
early FS6KD
middle FS6KD
late FS6KD
log2FC
K
early FS6KD
middle FS6KD
Ndufs3
Ndufs4
Ndufb8
Cox6a1
Atp5f1
Vinculin
L
early FS6KD
middle FS6KD
Cox6a1 Relative protein amount
Atp5f1 Relative protein amount
Ndufb8 Relative protein amount
Ndufs3 Relative protein amount
Ndufs4 Relative protein amount
ns

Fig. S4: ISR^{mt} is activated in middle-stage of FS6KD disease progression following *Atf3* induction.

(**A-D**) Expression shown as cardiomyocytes UMAP features of representative *Atfs* and ISR^{mt} genes (**A**) *Atf3*, (**F**) *Atf4*, (**G**) *Atf5*, and (**H**) *Mthfd2*.

(**E**) FA2 scatter plot colored by leiden cellular states and (**F**) PAGA plot colored by sample identity of PAGA trajectory from isolated cardiomyocytes.

(**G**) Proportion of each sample per leiden cluster within the main PAGA trajectory of the integrated dataset.

(**H**) Nuclear localization of Atf4 in 8-week FS6KD. Immunostaining with TnnI3 antibody (left), Atf4 antibody (middle; magenta), Hoechst (middle; blue) and an overlay image (right) (scale bar, 100 µm). A representative image was shown (early n=3, middle n=3, and late n=3 biological replicates).

(**I**) Quantitative analysis of Atf4 positive nuclei by immunostaining in FS6WT (n=4 biological replicates) and FS6KD of different stages (early n=3, middle n=3, and late n=3 biological replicates and n>3 technical replicates). Bars: mean ± SD. Dots: individual subjects. Ordinary one-way ANOVA and Tukey's test for multiple comparisons, statistical significance: not significant.

(**J**) Heatmap displaying expression levels of complex I and complex IV subunits and assembly factors ranked by log2FC.

(**K and L**) Protein analysis of selected mitochondrial complexes proteins. (**K**) Immunoblot analysis of complex I proteins Ndufs3, Ndufs4, Ndufb8, complex IV Cox6a1, and complex V Atpf1 in heart lysates from early (n=3 biological replicates) and middle-stage (n=3 biological replicates) mice and (**L**) quantification of Ndufs3, Ndufs4, Ndufb8, Cox6a1, and Atpf1 relative to early and normalized by loading control vinculin. Bars: mean ± SD. Dots: individual subjects. Student's *t*-test, statistical significance: $^{*}p \leq 0.05$, ns; not significant.
